# Folinic Acid Improves Healing of Diabetic Foot Ulcers

**DOI:** 10.1111/wrr.70141

**Published:** 2026-03-07

**Authors:** Glenn D. Hoke, Annjanette Stone, Michael A. Bauer, Weleetka C. Carter, Megan R. Newsom, Joseph V. Boykin

**Affiliations:** ^1^ Departments of Surgery/Plastic Surgery, Richmond Department of Veterans Affairs Medical Center Richmond Virginia USA; ^2^ Pharmacogenomics Analysis Laboratory, Research Service, Central Arkansas Veterans Healthcare System Little Rock Arkansas USA; ^3^ Department of Biomedical Informatics University of Arkansas for Medical Sciences Little Rock Arkansas USA; ^4^ Department of Surgery/Plastic Surgery Virginia Commonwealth University Health System Richmond Virginia USA

**Keywords:** diabetic foot ulcer, diabetic wound, DNA methylation, folinic acid, genomic, HMGB1, inflammation, microRNA, proteomic

## Abstract

A novel folinic acid (FA) wound treatment (FAWT) significantly (*p* < 0.05) improved healing (re‐epithelialization) of chronic diabetic foot ulcers (DFUs). In a double‐blind RCT, 10 (*n* = 10) chronic DFU subjects received daily topical treatment in either the Control group [(*n* = 5); PluroGel] or the FAWT group [(*n* = 5); PluroGel with FA 2.5%]. After 12 weeks, FAWT subjects experienced significantly (*p* < 0.05) greater %‐DFU area reduction (88% [SD: 16]) vs. Control (40% [SD: 39]), respectively. Proteomic analysis of keratinocytes (KCs) pre‐ and post‐FAWT, using Reverse Phase Proteomic Array (RPPA), documented significantly decreased levels of HMGB1 (High Mobility Group Box1) protein and activated IL‐1B protein at 12 weeks after FAWT as compared to Control. RPPA also documented significantly decreased activating phosphorylation of SAP/JNK3 (T183/Y185) and p38 MAPK (T180/Y182) levels after FAWT as compared to Control. These findings suggested decreased activation and possible reduction of NFKB/p65 and p38 MAPK following FAWT that could decrease proinflammatory gene expression. Genomic DNA‐methylation analysis of KCs identified significantly decreased FAWT‐induced methylation at gene expression regulatory sites for multiple microRNAs (MiRNA) associated with regulating proinflammatory responses. FAWT‐induced decreased methylation levels in MiRNAs suggested increased expression and their potential to inhibit protein translation. These MiRNAs are predicted to target multiple mitogen‐activated protein kinase (MAPK) pathways. These MAPK pathways mediate signalling from proinflammatory cell surface receptors (such as RAGE, TLRs and IL1R), altering gene expression. FAWT‐induced inhibition of MAPK‐signalling may lessen NFKB/p65 and p38 MAPK induction of proinflammatory gene expression, reflected in the significantly decreased protein levels of HMGB1 and IL‐1B. The data suggest FAWT‐induced coordinated expression of multiple anti‐inflammatory MiRNAs associated with impaired MAPK‐signalling that resulted in decreased expression of HMGB1 and IL1B and this process may facilitate DFUs' transition from an inflammatory state to wound repair, enabling KCs to regulate their proliferative phase, migration and the promotion of DFU re‐epithelialization.

## 1 Introduction

Impaired diabetic foot ulcer (DFU) healing is a significant and recurrent complication of diabetes. For the Type 2 diabetes mellitus (T2DM) population, DFUs affect 18.6 million people worldwide and 1.6 million in the United States each year [1]. The clinical settings of impaired DFU healing are frequently complicated by the absence of protective cutaneous sensation (diabetic neuropathy) and/or findings of chronic vascular insufficiency. If left untreated, 50% of chronic DFUs with impaired healing can progress to soft‐tissue infection (STI), diabetic foot osteomyelitis (DFO) and/or gangrene, where 20% of these patients will require hospitalisation. Between 15% and 20% of moderate to severe DFU infections or gangrene may lead to a lower‐extremity amputation (LEA). Individuals with DFUs have a 5‐year mortality rate of 30% that increases to greater than 70% with LEA. In the United States, more than 150,000 non‐traumatic LEAs associated with diabetes and/or DFUs are performed annually [1]. DFUs remain as the main cause of limb amputations worldwide, accounting for 80% of all non‐accidental amputataions [2]. Here, the absence of frontline interventions to promote effective chronic DFU healing, and a possible reduction in the numbers of infected or gangrenous DFUs requiring LEAs, remains a significant unmet need for our T2DM population.

Hyperglycemia induces dysregulation of timely progression through the healing process and inhibits keratinocyte (KC) capacity for migration and wound epithelialization, after injury The inflammatory phase of normal cutaneous wound healing is acute, spanning a few days, while in non‐healing chronic DFUs, inflammation becomes established and may impair wound resolution. In the normal acute wound bed, neutrophils are active in pathogen removal as well as release of proinflammatory molecules. Under conditions of increased proinflammatory stress, neutrophils may undergo NETosis, a programmed cell death in which networks of chromatin and antimicrobial proteins, called neutrophil extracellular traps (NET), are extruded into the extracellular space. Macrophages, which are involved in all stages of wound healing, from haemostasis through inflammation to tissue remodelling, also respond to the increased proinflammatory environment by releasing additional proinflammatory molecules. NETosis, along with the activation of neutrophils and macrophages, can lead to elevated levels of High Mobility Group Box 1 (HMGB1) protein. HMGB1 acts as a key extracellular proinflammatory signal that is linked to tissue damage and is associated with DFUs. Hyperglycemia also promotes the generation of Advanced Glycation End‐products (AGE), which act as proinflammatory ligands, binding and promoting increased inflammatory signalling, oxidative stress and cellular dysfunction through multiple cell surface receptors including RAGE, TLR2 and TLR4, and the TNF receptor, TNFAR [3].

Based on our earlier proteomic study measuring protein levels in KCs isolated from the debrided edge of either non‐healing or healing DFUs, we proposed that inhibition of chronic cellular inflammation could provide a positive benefit and lessen DFU severity [4]. From our proteomic DFU study, we observed: (1) significant increases in protein levels and the activation of pathways associated with KC proliferation and migration (PI3 Kinase and mTOR) and (2) evidence of increased cellular levels of proteins in non‐healing DFUs that suggested increased KC inflammation (increased COX2 expression). We also documented significantly (*p* < 0.05) decreased levels of cellular COX‐2 in KCs from healing DFUs, suggesting signalling through NFKB and p38 MAPK was being decreased. We also observed decreased levels of cell death‐associated proteins (p53, Bak and cleaved Caspase 9) in healing DFU KCs.

Subsequently, we provided a retrospective assessment of DFU healing in a cohort of chronic DFU‐patients administered high‐dose folic acid (HDFA; 5 mg/day with B6/B12 supplementation), while receiving standard wound care and off‐loading. We documented a post‐treatment relationship between HDFA administration and significantly decreased DFU‐wound areas, comparing untreated and HDFA‐treated subjects at 4 weeks before and following HDFA treatment (actual wound area reductions [0.5 vs. 5.4 cm^2^] and percentage of wound closure [−2% vs. 72%], *p* < 0.05; respectively) [5]. Data from this study provided evidence of accelerated DFU closure and complete healing for the HDFA group. This retrospective assessment supported a premise that impaired signalling and healing of chronic DFUs resulted from hyperglycemia‐induced proinflammatory changes inhibiting KC proliferation and migration, that are necessary for wound closure.

The current, randomised, proof‐of‐concept (POC) trial was designed as an integrated study of genomic and proteomic data from DFU KCs to elucidate mechanisms of DM and folate supplementation that affect epigenetic processes and cellular signalling pathways associated with the impaired healing of chronic DFUs [6, 7]. The hypothesis of this study is that a folate (folinic acid) supplemented topical treatment for chronic, non‐healing DFUs may facilitate improved KC migration, proteogenomic signalling and wound closure that may decrease the significant morbidity experienced by subjects with T2DM and chronic DFUs. As it affects the alteration of epigenetic processes, folate supplementation may decrease chronic DFU inflammation, MiRNA‐mediated target suppression or upregulation and MAPK inflammatory signalling [8]. These processes may facilitate the inhibition of excessive inflammatory cytokine production (e.g., HMGB1) and enhance the recovery of normal post‐injury KC functions and DFU re‐epithelialization. With validation from this POC study, the novel FAWT could provide clinical value in the frontline management of chronic DFUs.

## 2 Materials and Methods

### 2.1 Clinical Trial Design

This clinical trial was performed at the Richmond VAMC Outpatient Wound Healing Clinics, Richmond, VA. The study protocol and consent forms meet the CONSORT criteria and were approved by the Richmond VAMC Institutional Review Board (IRB). This study is registered with IRBNet (ID# 15272843) and ClinicalTrials.gov (ID: NCT04723134). The study was conducted in accordance with the Declaration of Helsinki (1975) and the Belmont Report (1979) as related to the ethical principles and guidelines for research involving human subjects. The study involved human subjects who gave prospective written informed consent and demonstrated their understanding.

A double‐blind randomised controlled trial (RCT) was designed for this study to evaluate the efficacy of a novel topical FAWT for the management of DFUs. DFU study subjects were adult Veterans with type II diabetes and chronic (> 4 weeks) full‐thickness, neuropathic DFUs selected from the patient population of the Richmond VAMC Outpatient Wound Healing Clinic (WHC). A total of 10 (*n* = 10) Veteran DFU‐wound study candidates (SCs) were selected from the consented volunteers and randomised for Control (*n* = 5) or FAWT (*n* = 5) study groups (see Table 1).

**TABLE 1:** Profiles for DFU study subjects treated with PluroGel (Control) or PluroGel with 2.5% folinic acid (FAWT).

Age (years)	Gender	Tobacco use	BMI	Diabetes meds	Hgb A1C	DFU location	DFU area (cm^2^) (D)	DFU age (days)	ABI/TBI Toe (mmHg)	LEA^a^	Days to heal^b^	% Closure at 12 weeks^c^	Healed (H) or unhealed (UH)^d^
Control													
69	M	N	29.3	None	6.8	Right lateral ankle	0.83 (0.3)	168	1.4 0.86 91	N	36	100	H
72	F	Y	34.2	Insulin	6.6	Left lateral foot	3.37 (0.2)	28	0.99 0.70 139	N	NA	50	UH
73	M	N	34.3	None	6.8	Left great toe	0.56 (0.3)	184	1.45 ++ ++	N	NA	0	UH
73	M	N	25.7	Insulin	6.5	Right heel	3.81 (0.4)	899	++ 0.27 47^c^	N	NA	12	UH
70	M	N	21.1	Insulin	6.8	Left plantar foot	1.78 (0.2)	1259	1.25 ++ ++	Y L/toe	NA	40	UH
FAWT													
72	M	N	30.5	Metformin	6.9	Left lateral foot	5.52 (0.4)	730	++ 1.2 115	N	80	100	H
62	M	N	35.8	Metformin	6.6	Right medial ankle	5.64 (0.4)	161	1.34 0.95 127	N	NA	60	UH
82	M	N	28.5	Insulin Linagliptin	7.4	Right lateral foot	0.97 (0.2)	339	++ 0.35 59	N	145	93	H^d^
80	M	N	36.4	Metformin Glipizide	7.7	Left great toe	0.54 (0.1)	168	1.25 1.25 182	N	160	97	H^d^
71	M	N	21.1	Insulin	6.8	Left plantar foot	1.43 (0.2)	1609	1.25 ++ ++	Y L/toe	N/A	80	UH

Abbreviations: ABI, ankle brachial index; BMI (kg/m^2^), body mass index; DFU, diabetic foot ulcer; FAWT, folinic acid wound treatment; LEA, lower extremity amputation; TBI, toe brachial index.

^a^ LEA, history of lower extremity amputation, BKA, below the knee amputation, Y, yes, N, no, with location of LEA.

^b^ Days to full wound healing following treatment with PluroGel alone (Control group) or PluroGel with 2.5% folinic acid (FAWT group).

^c^ Percent of DFU area closed by 12 weeks.

^d^ Wound closed (H) by 16 weeks following start of study.

The assessment and implementation of study DFU offloading was provided by the VAMC Podiatry Clinic and was required for all study subjects. During DFU evaluation, forefoot and midfoot DFUs were offloaded using a controlled ankle motion (CAM) walker, which significantly reduces peak plantar pressure by limiting ankle joint range of motion. For rearfoot DFUs, offloading was achieved with a CAM boot in conjunction with a knee scooter, which prevented weightbearing on the affected foot while simultaneously restricting motion. In cases where patients exhibit instability with ambulation in a CAM walker or were unable to utilise a knee scooter, a Darco postoperative shoe was employed. Appropriate dispersion padding was affixed to the insole to optimise pressure redistribution and enhance offloading.

A double‐blind randomised process of DFU‐wound treatment was provided and maintained by the Research Pharmacy for the assignment of Control or FAWT treatment to consecutive, consented study subjects. Study subjects, the principal investigator (PI) and the research investigation medical team remained blinded to the study subject treatment assignments during the clinical trial. The study treatments (Control and FAWT) were provided to the randomly assigned subjects in single 1 oz. White Ointment jars, with screw on lids, to hold 50 g of the assigned treatment. Topical wound treatment was performed by using sterile tongue blades to apply the treatment (dime thickness) daily to the Vashe‐cleansed study wound, with cotton gauze dressing secured with tape and or Tetra‐Net. Weight of the closed study treatment jar (gm) was obtained weekly during clinic evaluations. PluroGel (Medline Industries) was provided for use as a Control treatment or compounded with Folinic acid calcium salt (Leucovorin; calcium folinate) to provide a 2.5% (molar weight) mixture with PluroGel, provided as the FAWT. The compounding pharmacy provided identical jars of labelled Control or FAWT treatments for randomised study subject assignments that were indistinguishable. Study subjects were provided with new jars of their assigned treatment every 2 weeks. All treatment jars were kept refrigerated by study subjects. At the completion of 12‐weeks of wound treatment, or after complete wound closure, identification of the blinded DFU treatment (i.e., Control or FAWT) was provided by the Research Pharmacy.

### 2.2 Study Candidate (SC) Inclusion Criteria

- DFU criteria for enrollment. A SC must be observed with: (1) at least a 4‐week history of a non‐healing (< 50% wound area closure/4‐weeks), superficial DFU (PEDIS‐Grade 1 or 2 Depth [wound above fascia without infection, exposed tendon or bone]); (2) initial, chronic DFU‐wound area between 0.5 and 12 cm^2^, without evidence of advancing wound closure for 4‐weeks; (3) DFU‐wound with any appreciable level of clinical neuropathy (i.e., SWME or superficial pain test); (4) lower extremity non‐invasive Doppler studies of DFU‐lower extremity with ABI value ≥ 0.7 and/or toe pressure ≥ 50 mmHg and (5) chronic DFU without evidence of acute infection (e.g., cellulitis, abscess or osteomyelitis) or microbial colonisation (> 10^4^ CFU/g); determined at the time of enrollment.
- SC may present with multiple DFUs; however, only one DFU per SC would be selected for study. Preferably, the largest DFU observed will be picked for study.
- If the SC is a woman of childbearing potential, she must be practicing an acceptable form of birth control, as determined by the Investigator. All females with an intact uterus must have a negative Beta‐HCG test (< 5 mIU/mL) to proceed with study participation.

### 2.3 Study Candidate Exclusion Criteria

- Any SC who is unable to provide written informed consent will not be enrolled for study participation.
- Any SC who is pregnant or breastfeeding will not be allowed to enrol or continue in the study, as pregnancy or breastfeeding may impact the study results.
- SC with DFU less than 0.5 cm^2^ or greater than 12 cm^2^ will be excluded from the study.
- SC who are unable to comply with the recommended off‐loading of the selected DFU will be excluded from the study.
- Any SC with a current active history of alcohol or substance abuse will be excluded from study participation.
- Any SC receiving steroid therapy (prednisone), chemotherapy or biological therapies (e.g., Humira) within 90‐days will be excluded.
- SC with ABI values < 0.7 or toe pressure < 50 mmHg, for the involved lower extremity of the study DFU, will be excluded from study participation.
- SC with a HgbA1C > 9 will be excluded from study participation.
- Any SC with a medical condition which, in the opinion of the investigator, should exclude him/her from participating in the study.

### 2.4 Treatment and Sample Collection

During the study, two sets of adjacent, 3 mm punch biopsy specimens were obtained from the viable margins of the study DFU. Full‐thickness, adjacent marginal specimens were obtained at the beginning of randomised study treatment and again after 12 weeks of treatment or after complete healing of the study DFU. If needed, study subjects were allowed to receive injectable local anaesthetic for tissue retrieval when punch biopsy specimens were obtained. Following punch tissue retrieval, tissue specimens were selected, stored and prepared for proteomic (P#1) or genomic (P#2) studies. P#1 specimens for proteomic analysis were embedded and frozen in OCT compound immediately after retrieval and stored at −80°C. P#1 biopsies were microscopically verified to be DFU KCs suitable for laser‐capture microdissection (LCM), to support lysates for microarray slide preparations. The second punch specimen (P#2), retrieved for genomic studies, received epidermal/dermal layer separation using a heated‐water bath method [9]. P#2 epidermal specimens were frozen and stored in cryovials at −80°C for genomic analysis and DNA isolation for methylation studies.

### 2.5 Study Wound Imaging

The initial pre‐treatment and successive post‐treatment study DFU areas (cm^2^) were obtained using the MolecuLight i:X Wound Imaging Device. MolecuLight digital images and fluorescent microbial scanning were employed routinely during this study to document study DFU areas (cm^2^) and to provide targeted sampling for identification of microbial colonisation (≥ 10^4^ CFU/g), respectively.

### 2.6 Proteogenomic Laboratory Analysis

Quantitative measurements of proteins were performed using a multiplexed, immuno‐proteomic microarray platform, reverse phase protein array (RPPA), to determine the cellular levels of specific proteins or activating levels of protein phosphorylation states. Protein levels between pre‐ and post‐treatment groups for Control (*n* = 4) and FAWT (*n* = 4) were used to determine levels of differential expression. Here, one biopsy from an FAWT‐treated subject and one biopsy from a Control‐treated subject did not provide sufficient total protein to support RPPA analysis (FAWT, *n* = 4 and Control, *n* = 4). The LCM and RPPA were provided by the Center for Advanced Proteomics and Molecular Medicine, George Mason University, Manassas, VA. Genomic data documenting CpG DNA methylation states and microRNA methylation patterns, comparing pre‐ and post‐treatment Control and FAWT specimens, was performed using Infinium Methylation Analysis provided by the VA Pharmacogenomics Analysis Laboratory, Research Service, Little Rock, AR.

### 2.7 RPPA Analysis

Quantitative measurements of proteins using RPPA were performed following processing of fresh‐frozen biopsies. RPPA provides a high‐throughput procedure capable of simultaneously analysing hundreds of samples automatically arrayed in replicates. RPPA measures the intensity of arrayed lysates (approximately 10,000 cells per experimental condition onto multiple arrays) and interrogates them with a primary, specific antibody and a secondary, signal amplification antibody, allowing for the quantitative measurement of each individual protein, with data normalised to the total protein per spot. RPPA allows for the detection of total protein (representing final state of expression/translation) and post‐translation phosphorylation to monitor the activation or silencing of signalling pathways.

Each antibody‐specific slide provides a quantitative measurement of individual proteins and levels of activating protein phosphorylation. Enriched KC populations were isolated by laser capture frozen biopsy tissue (see above), capturing approximately 10,000 epithelial KCs from each sample. Micro‐dissected material was stored at −80°C and prior to RPPA array deposition, samples were lysed in extraction buffer composed of Tissue Protein Extraction Reagent (TPER; ThermoFisher), 2× SDS‐PAGE Sample Buffer (ThermoFisher) mixed 1:1 and 2.5% beta‐mercaptoethanol (BME) to a final lysate protein concentration of approximately 250 μg/mL in extraction buffer. Collected KC samples were heated at 100°C for 5 min, brought to room temperature, briefly centrifuged and then stored at −80°C until ready for printing. Lysate protein concentrations were determined and deposited using an Aushon automated sample arrayer on replicate microarray slides. Semi‐automated probing of arrayed slides with validated (for specificity, linearity and dynamic range) antibodies allows for each protein target to be measured quantitatively with onboard replicates and performance measured internal controls [10]. Each replicate slide is interrogated with a pre‐validated primary antibody, specific to the proteins or post‐translationally modified proteins of interest, allowing all samples to be processed simultaneously, limiting potential variability. Signalling is amplified through a secondary detection antibody that allows for a quantitative measurement of the protein in the deposited lysate using laser scanning and commercial automated data analysis.

For this study, 40 different proteins and modified proteins (Table 2) were analysed by RPPA to assess the cellular levels and post‐translational modification (PTM)‐induced changes in the signalling architecture present in KC lysates. RPPA analysis provided the means to assess changes in protein networks and monitor the impact of FAWT in DFU KCs on cellular pathway signalling. Unique RPPA antibody identifiers were mapped to UniProt protein accessions and HGNC (HUGO Gene Nomenclature Committee) identifiers through manual inspection of commercial antibody names, specificity and corresponding human protein entries curated within the UniProt resources (http://www.Uniprot.org).

**TABLE 2:** Total protein or post‐translation modified‐protein levels quantitatively measured using RPPA.

Total protein	Post‐translation modification
4EBP1	AKT (P‐S473)
BAK	AKT (P‐T308)
Gasdermin D	CREB1 (P‐S133)
HMGB1	eNOS (P‐S1177)
IL‐6	Ikapp‐a (P‐S32/S36)
IL‐8	IKK‐g (P‐S376)
IRAK‐2	IL‐1B Cleaved D116
MMP9	IRF3 (P‐S386)
MyD88	MDM2 (P‐S166)
p85a	NFKB/p65 (P‐S529)
PI3K	p38 MAPK/MAPK14 (P‐T180/Y182)
PIP2	p53 (P‐S15)
RAGE	p90RSK/RPS6Ka1 (P‐380)
SMAC/Diablo	PDK1 (P‐S241)
SOX‐2	ROS (P‐Y2274)
TIRAP	SAPK/JNK3/MAPK10 (P‐T183/Y185)
TLR‐4	SEK1/MAP2K4 (P‐S78)
TLR‐9	SGK1 (P‐S78)
TNF‐a	TAK1/MAP3K7 (P‐S412)
TRAF6	TBK1/NAK (P‐172)

### 2.8 Genomic DNA Methylation Analysis

DNA was extracted from frozen epidermal tissue (see above) as described in the DNeasy Blood & Tissue Kit (Qiagen, Germany, Cat No. 69504) according to manufacturer's protocol. Tissue specimens greater than 25 mg were sectioned into small tissue segments to ensure efficient lysis. Margins of the tissue were preserved after full‐thickness trimming. Following Proteinase K treatment, tissue samples were heated at 56°C for complete lysis. DNA was eluted in 25 μL of buffer AE. DNA was quantitated with RNaseP fluorescence using Quant Studio 12 k Flex quantitation system. Sample quality was determined from O.D. ratios obtained with Nanodrop 8000 Spectrophotometer. Bisulfite modification was performed with Zymo EZ‐96 DNA Methylation‐Lighting kit (Cat No. D5033) according to manufacturer's instructions. A further assessment of bisulfite‐mediated conversion efficiency was determined with DAPK1 PCR primers (IDT Technologies) and gel electrophoresis of PCR products. Bisulfite‐modified DNA (min. input 500 ng) was whole‐genome amplified, hybridised to Infinium MethylationEPIC v2.0 microarrays, single‐base extended and stained using the Automated Protocol for the Illumina Infinium HD Methylation assay. GenomeStudio (GS) software (version 1.9.0) and BeadArray Control Reporter (version 1.1.0) freeware was used to assess internal microarray quality control. These data were analysed with background subtraction, no normalisation. All samples met internal quality control specifications for sample‐dependent and sample‐independent controls. Replicates had 100% correlation using built in sample identity SNPs (*n* = 65 non‐CpG probes and all samples had detected CpG (0.0.1) greater than 99% each determined suitable for further bioinformatic analysis).

### 2.9 Methylation Data Analysis

The R package CpGAssoc [11] was used to identify significantly differentially methylated probes (DMPs) through fixed effect models, assessing associations with FAWT. Additionally, the R package SeSAMe [12] was employed for both DML (differential methylation locus) and differentially methylated region (DMR) analyses, which internally utilise the mixed linear model framework for DML analysis and merges neighbouring CpGs that show consistent methylation variation for DMR detection. All analyses were performed using normalised (quantile normalisation) and batch‐corrected beta values as input. Race was included as a covariate to control for potential confounding effects and results were considered significant with adjusted *p*‐values < 0.05. To investigate the association of methylation status and biological relevance, a gene set overrepresentation analysis (ORA) was conducted using the web tool NetworkAnalyst [13]. NetworkAnalyst web‐interface was used to visualise the protein–protein interactions of gene annotated significant DMR near genes. The PPI networks were derived from the IMEx interactome database. A 1st order network was selected which allowed the addition of higher‐order interactions from the database. We then performed the gene set ORA on the resulting PPI network using the Reactome pathways. The pathways that were significant, with false discovery rate (FDR) ≤ 0.05, were retrieved.

### 2.10 MiRNA and mRNA Target Prediction Analysis

These analytical tools were used to identify MiRNAs that have a high probability of targeting potential mRNAs and their associated genes with filtering, to identify those associations exhibiting a confidence level of 90% or greater. The general specifics for the programmes are listed below:

#### 2.10.1 miRDB

An algorithm‐based database for MiRNA target prediction using a bioinformatics programme. miRDB analyses mRNAs against MiRNA seed hairpin sequences using data from high‐throughput sequencing experiments. Pairings are based upon a scale of 100 (Highest)– 50 (Lowest), as below 50 miRDB these are potentially unstable candidate pairings. Data reported in this manuscript relied on a score of 90 or higher [14, 15].

#### 2.10.2 TargetScan

An algorithm‐based prediction programme that identifies potential mRNA targets based on MiRNA‐seeded sequence, structures and target mRNA characteristics. Scoring is based upon the conservation of nucleotides in the mRNA complementarity to the seed sequence in MiRNAs. Results are based upon a scale of 1.0 (Highest) to 0 (Lowest). Data reported in this manuscript relied on a score of 0.90 or higher [16].

#### 2.10.3 DIANA‐mirPath

Set of tools for prediction of MiRNA targets (in CDS or 3′‐UTR regions) provided by the DIANA‐microT‐CDS algorithm or even experimentally validated MiRNA interactions derived from DIANA‐TarBase. These interactions are based upon experimentally validated (micro‐array. proteomic and sequencing) data to identify MiRNA‐target pairs. TarBase scales MiRNA:Target mRNA/Gene data generating an Interaction Score with a scale of 1.0 (Highest) to 0 (Lowest) [17].

#### 2.10.4 MiRTargetLink

An analysis and visualisation interface to explore and analyse interaction networks between MiRNAs and target genes from other MiRNA prediction programme's databases. MiRNA:Target interactions can be sorted by levels of (strong or weak support) and results are Scored from 1.0 (Highest) to 0 (Lowest) degrees confidence. Data reported in this manuscript relied on a score of 0.90 or higher [18].

### 2.11 Statistics

Statistical analysis for comparative summary measurements or analysis for clinical wound parameters and protein expression (analyte) comparisons, with RPPA, was performed using GraphPad Prism Version 10.6.1 (799), September 2025 (GraphPad Software Inc., San Diego, CA). The level of significance was taken as *p* < 0.05 and results are expressed as mean ± standard deviation (SD).

## 3 Results

### 3.1 Clinical Response

**FIGURE 1:**
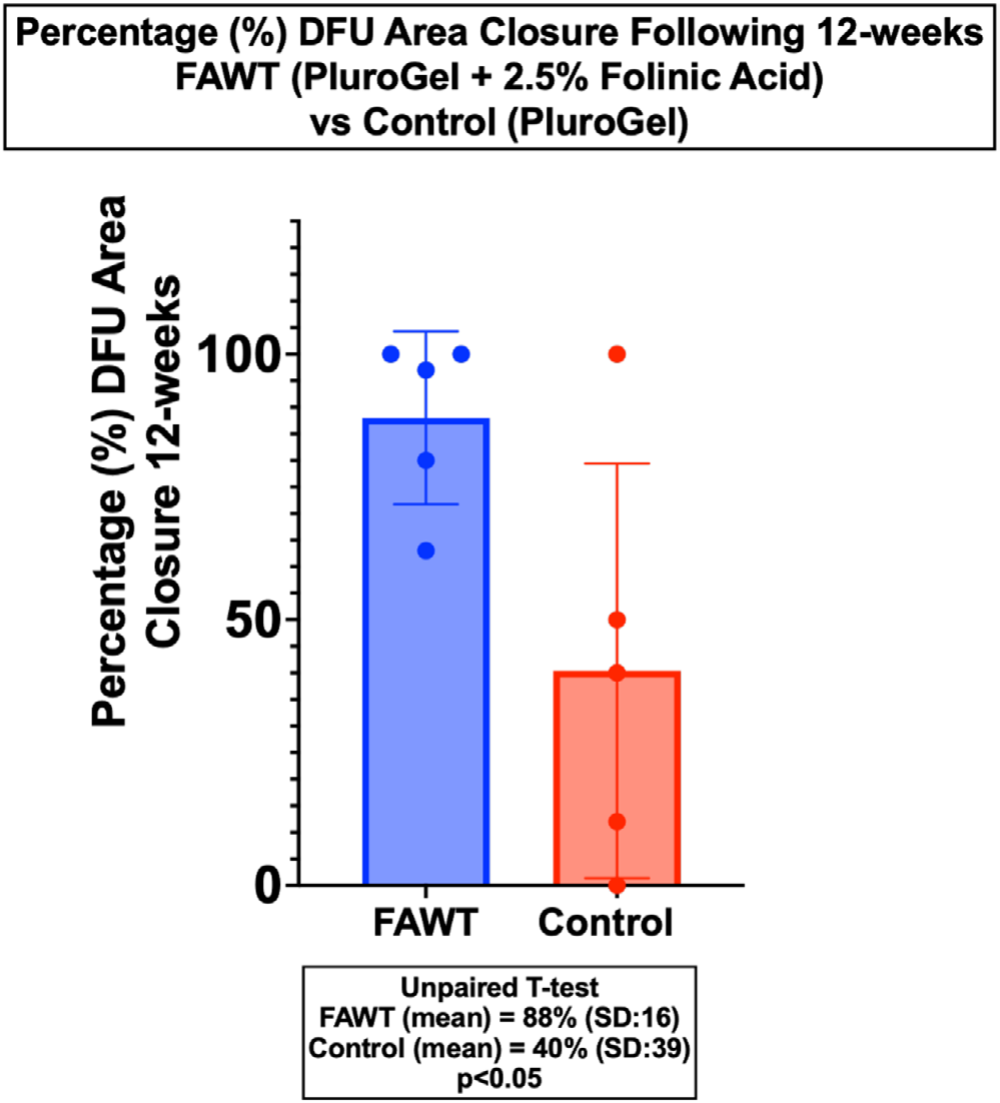
Percentage closure of DFUs observed following 12 weeks of daily topical FAWT (*n* = 5) versus Control (*n* = 5). FAWT‐mean area closure = 88% (SD: 16); Control‐mean area closure = 40% (SD: 39); *p* < 0.05 (unpaired *t*‐test).

**FIGURE 2:**
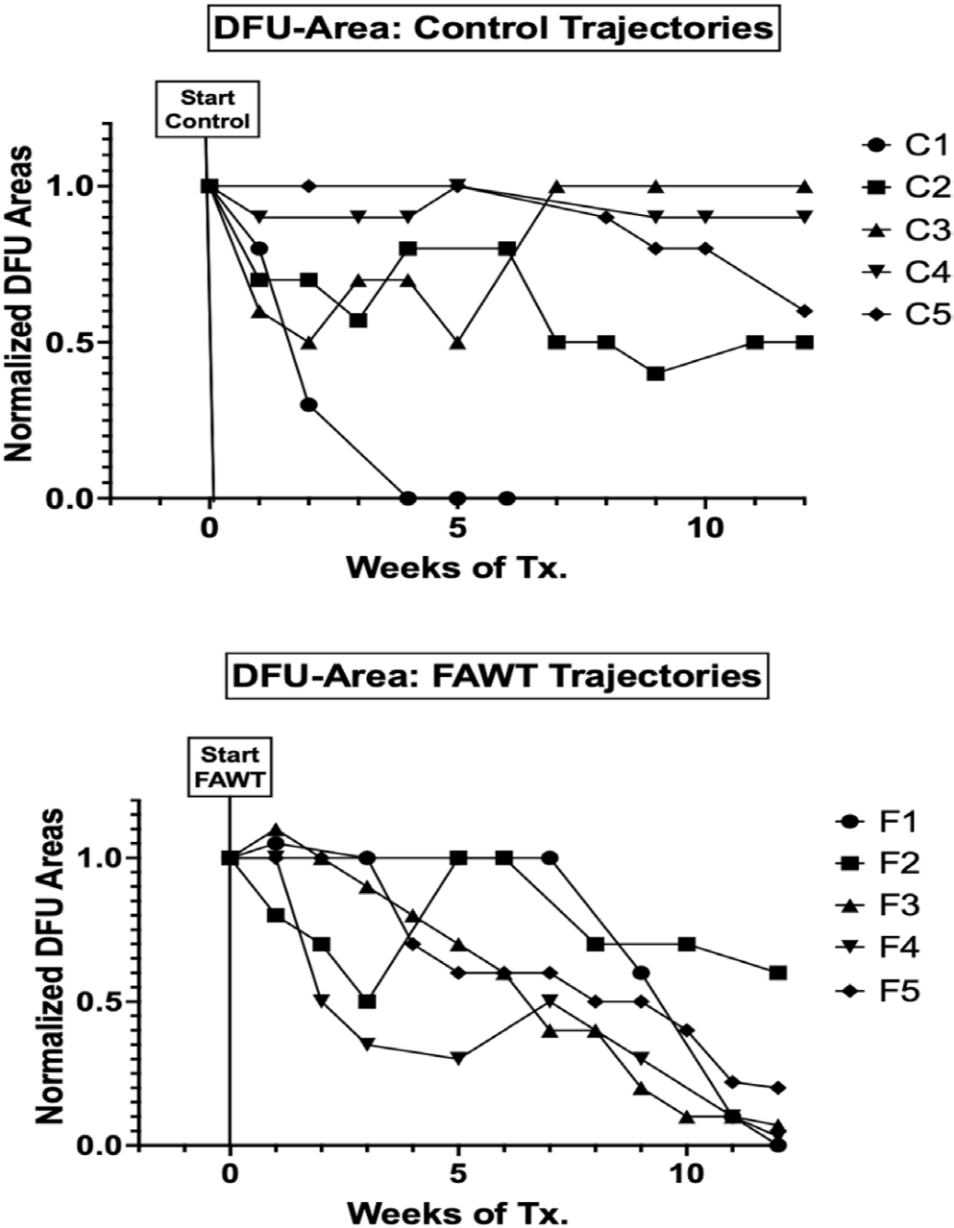
DFU normalised area trajectories following randomised Control (upper graph) or FAWT study treatment (lower graph). Data points obtained during routine DFU study evaluations. See Table 1 for corresponding study subject profile and initial DFU areas (cm^2^).

The current study was designed to evaluate a topical folinic acid (2.5% folinic acid calcium salt) preparation (FAWT) for its ability to promote healing and closure of DFUs. Chronic DFU subjects (*n* = 10) were randomly assigned to 12 weeks of daily topical wound treatment with either Control (*n* = 5, PluroGel, Medline Industries) or FAWT (*n* = 5, PluroGel + 2.5% folinic acid calcium salt). DFU closure outcomes for Control and FAWT subjects were tracked following 12 weeks of treatment. At the end of 12 weeks, DFU subjects receiving FAWT experienced significantly (*p* < 0.05; unpaired *t*‐test) greater %‐DFU area closure compared to Control (88% [SD: 16] vs. 40% [SD: 39]), respectively (Figure 1). Wound healing trajectories are provided from study subject DFU evaluations performed at 1–2 week intervals (Figure 2). Trajectory data provide normalised changes in DFU areas from the start of randomised Control and FAWT study treatment for 12 weeks.

The initial (pre‐treatment) mean DFU areas (cm^2^) were obtained using the MolecuLight digital imaging system and GraphPad Prism10 statistical software (see Table 1). Starting DFU group mean areas (SD) were 4.0 cm^2^ (± 2.0) for the FAWT group (*n* = 5) and 2.5 cm^2^ (± 0.9) for the Control group (*n* = 5). Group comparisons were obtained with the unpaired *t*‐test with Welch's correction (*t* = 0.6732; df = 5). A two‐tailed *p*‐value of 0.53 was obtained. While the mean DFU area was larger for the FAWT group, there was no significant (*p* = NS) difference between the FAWT and Control mean wound areas at the start of randomised DFU study treatments.

### 3.2 Proteomic Analysis

**FIGURE 3:**
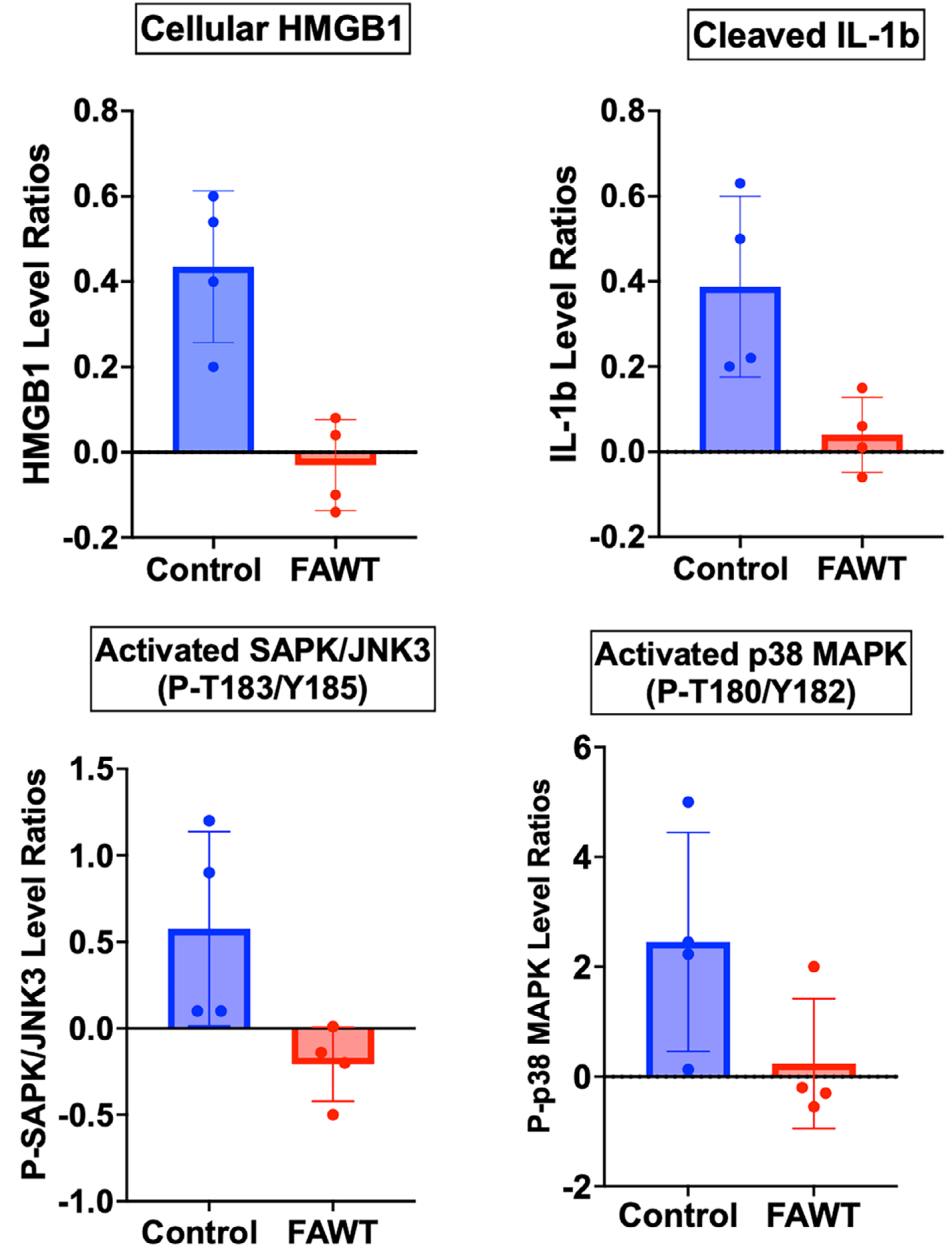
Illustration of DFU‐keratinocyte differential analysis ratios of HMGB1, IL‐1B, SAPK/JNK and p38 MAPK for Control and FAWT study groups. Significantly (*p* < 0.05) decreased ratios of proteins measured by RPPA from pre‐ and post‐treated protein levels demonstrate that FAWT‐induced decreased cellular levels in HMGB1 and cleaved IL‐1B along with decreased levels of phosphorylated SAPK/JNK3 and phosphorylated p38 MAPK. All analyses performed with *n* = 4, for both Control and FAWT groups (see Section 2.6).

The multiplexed, quantitative proteomic assessment of LCM‐captured KC provided data on the signalling architecture in DFU KC. While there were no significant changes in protein levels with Control treatment, there was evidence of suppressed mitogen‐activated protein kinase (MAPK) signalling in response to FAWT. Using differential expression analysis (DEA), FAWT subjects exhibited decreased levels of activating phosphorylation in SAPK/JNK3 (MAPK10) at T183/Y185 and in p38 MAPK at T180/Y182 (Figure 3). The FAWT group also exhibited significantly (*p* < 0.05; unpaired *t*‐test) decreased intracellular levels of HMGB1 and activated (cleaved) IL‐1B. The RPPA data suggests that FAWT resulted in a lessening of proinflammatory signalling through MAPK pathways, which repressed the expression of HMGB1. The observed decreased level of cleaved IL‐1B, also regulated by MAPKs, may be the result of decreased expression and/or increased extracellular export.

**FIGURE 4:**
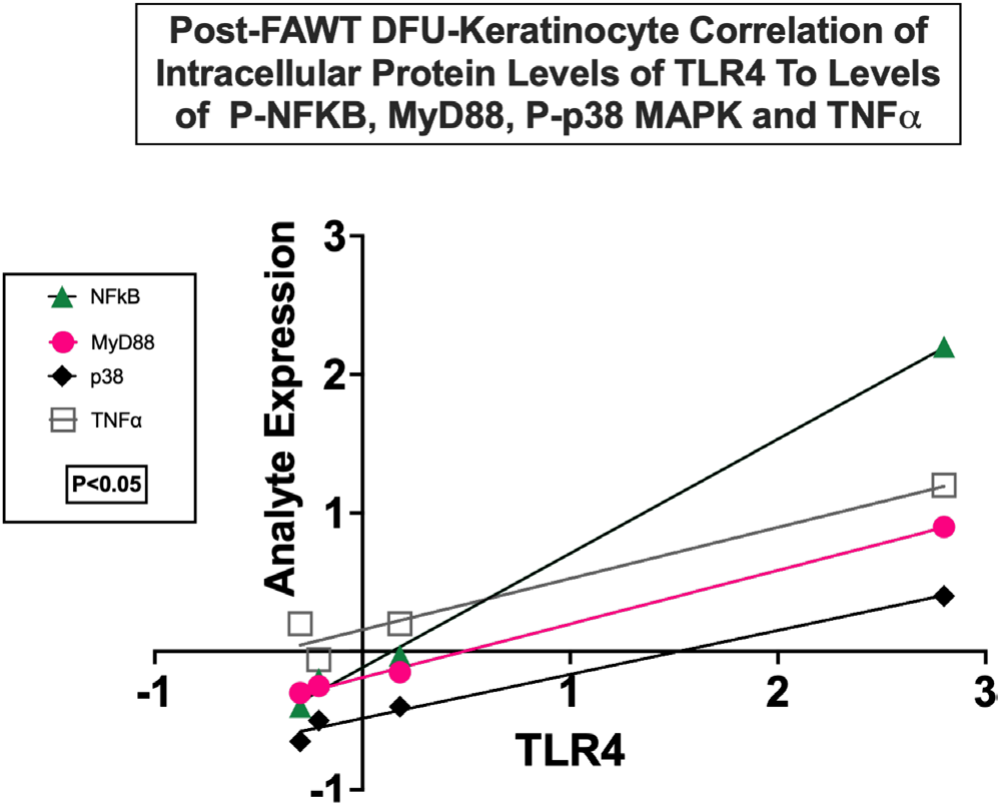
Correlation (*p* < 0.05; Unpaired t‐test) of TLR4 inflammatory pathway signalling protein expression following FAWT DFU‐treatment was documented, using differential expression analysis, for NFKB, MyD88, p38 MAPK and TNFα. These observations suggest the presence of MyD88‐dependent TLR4 downstream signalling. Correlation for TLR4 was not observed for MyD88, NFKB, p38 MAPK or TNFα following Control treatment. Proteomic study groups: FAWT (*n* = 4) and Control (*n* = 4) (see Section 2.6).

Additionally, the cellular levels of TLR4, a major regulator of immune responses, exhibited correlations when compared with the expression levels of MyD88, p38 MAPKs, NFKB/p65 and TNF‐α (Figure 4). The TLR4/MyD88/NFκB axis is an important signalling pathway in innate immunity and inflammation. The correlation data for TLR4 suggests that inflammatory signalling through p38 MAPK may also be involved in a FAWT‐induced decrease in this pathway in DFU KC. Dysregulation and increased signalling through this pathway appear to play a major role in promoting DFU healing. Ligand binding and activation of TLR4 initiates the MyD88‐dependent downstream activation of p38 MAPK and subsequently NFkB expression of transcription factors that control proinflammatory gene expression [19].

The TLR4/MyD88‐complex initiates signalling through MAPKs to regulate p38 MAPK and NFKB activity. The post‐FAWT relationships between TLR4 and downstream gene expression correlate with increased healing of DFU [20, 21]. Depressed inflammatory signalling through these pathways in healing wounds following FAWT is supportive of our earlier observation of lower COX‐2 in healing DFU KC [4]. The COX‐2 promoter contains binding sites for several transcription factors (including: SP1, ELK1, STAT3, CREB1, SP3 and EGR2) that are regulated by MAPK signalling and associated with ERKs, p38 MAPKs and JNKs activation [22, 23].

FAWT decreased activation of SAPK/JNK3 and p38 MAPK, which correlated with decreased activation of signalling through TLR4/MyD88 to activate MAPK pathways in response to decreased proinflammatory signalling from cell surface receptors. Decreased signalling through MAPKs, as indicated by decreased activating‐phosphorylation levels of JNK3 and p38 MAPK, suggests a lessening of the downstream activation of gene expression resulting from MiRNA inhibition of p38 MAPK and NFKB/p65 [24]. These MAPK‐pathways are interconnected by cross‐talk between the individual MAPKs, linking extracellular receptor activation to chronic inflammation as observed in DFU. The correlation analysis (*p* < 0.05; unpaired *t*‐test) between TLR‐4, a receptor mediating HMGB1 signalling, and the expression levels of NFKB/p65, p38 MAPK or MyD88 (an innate immune signal adaptor transduction protein) suggested that signalling through MyD88‐associated cell surface receptor activation was occurring in DFU KC (Figure 4). The correlation with MyD88, required for TLR4 and IL‐1R (IL‐1B receptor) signalling, suggests that these pathways may be involved in the increased expression of HMGB1 and perhaps IL‐1B [25]. These correlations lend support to the hypothesis that FAWT reduces proinflammatory signalling and the synthesis of IL‐1B and HMGB1 by KC. The reduction of inflammation in KC, and perhaps other cells in the wound bed (neutrophils and macrophages), may lower the overall proinflammatory environment, limiting inflammatory signalling through cell surface receptors (TLRs, IL1R) on KC. The impact on KCs is to lessen signalling through MAPKs and reduce the synthesis and release of HMGB1 and IL‐1B, with downstream activation of gene expression by NFΚB/p65 and perhaps p38 and ERK1/2. These pathways are connected by MAPK signalling cascades that link extracellular receptor activation to chronic inflammation observed in DFUs [26].

### 3.3 DNA Methylation

Altered DNA methylation has been documented to induce changes in MAPK‐signalling cascades, enforcing the potential regulatory roles of folates in immune and inflammatory responses [27]. The Infinium MethylationEPIC v2.0 BeadChip data exhibited significantly increased or decreased methylation at regulatory sites containing CpG dinucleotides following 12 weeks of FAWT.

Depending on the location and the methylation status of these CpG dinucleotides, methylations may exert positive or negative regulation of gene expression. The DNA methylation platform interrogates chromosomal sites associated with transcription initiation, enhancer binding, polycomb binding or other potential MiRNA regulatory regions. Also, methylation can result in chromatin remodelling and histone sliding which may expose DNA that is not methylated, increasing unscheduled transcriptional activation [28].

Changes in pre‐ and post‐FAWT DNA methylation levels that increased or decreased by greater than 20‐fold were considered candidates for further analysis.

**FIGURE 5:**
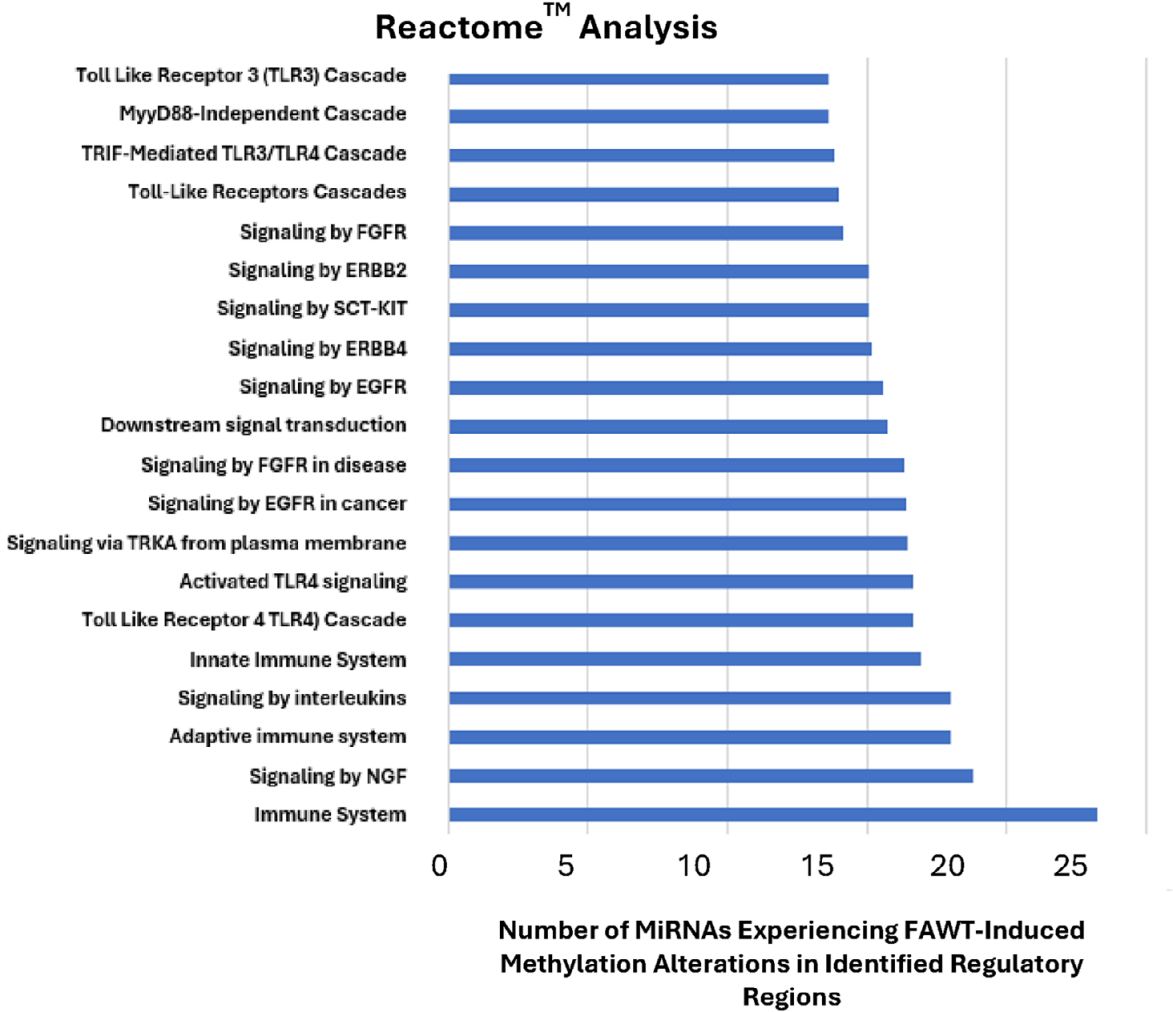
Reactome analysis showing the Top 20 signalling pathways impacted by FAWT based upon the number of MiRNAs that experience significantly altered levels of methylation in identified regulatory regions. Many of these relate to modulation of the immune response, signalling through TLR‐driven pathways and signalling pathways from growth receptors.

The differential methylation analysis identified decreased methylation in important regulatory sites located in multiple MiRNA genes. In the pre‐ and post‐FAWT data, of the MiRNA sites identified as having altered methylation levels, 208 exhibited greater than a 20‐fold increase in methylation and 403 exhibited less than a 20‐fold decreased methylation. Many of these MiRNAs were predicted to target MAPKs which are known to regulate proinflammatory signalling (see Supporting Information Tables A–G). Focusing on MiRNAs due to their roles as epigenetic regulators of protein expression/translation, FAWT treatment exhibited increased and decreased methylation states in regulatory sequence sites. These MiRNAs were subjected to Reactome‐based association analysis to identify potential pathways that were affected (Figure 5). Reactome analysis of the MiRNA data obtained from FAWT KC indicated Toll Receptor Signalling, MyD88‐mediated receptor‐signalling (potentially involving TLRs, RAGE, EGFR, etc.) and proinflammatory signalling were most prevalent [29].

These major pathways support a potential role for FAWT in the depression of MAPK signalling that may be activated by multiple cell surface receptors, including RAGE, TLRs, TNFRs or IL1Rs and activation of p38 MAPKs, which induce the expression of proinflammatory transcription factors. FAWT appeared to coordinate a decreased methylation of regulatory sequences in several MiRNAs, with some being targeted by multiple MiRNAs, which increases the likelihood of impacting specific MAPKs (Table 3).

**TABLE 3:** Identified MAPKs targeted by validated MiRNAs exhibiting less than 20‐fold decreased CpG‐methylation in regulatory regions that modulate gene expression.

MAPK	Targeting MiRNA	MAPK	Targeting MiRNA
MAPK4	MiR‐2682‐3p	MAP2K4	MiR‐27b‐3p
			MiR‐23b‐3p
MAPK14	MiR‐27b‐3p		
	MiR‐2682‐3p	MAP2K6	MiR‐8085‐3p
			MiR‐7‐2‐3p
MAP3K9	MiR‐2682‐3p		MiR‐9‐3‐3p
			MiR‐205‐3p
MAP3K5	MiR‐27b‐3p		MiR‐1298‐3p
ASK1	MiR‐23b‐3p		MiR‐186‐3p
	MiR‐7‐2‐3p		MiR‐4763‐3p
	MiR‐205‐3p		
	MiR‐106b‐3p	RPS6KA1	MiR‐93‐3p
		(RSK1)	
MAP3K4	MiR‐27‐3p		
		RPS6KA5	MiR‐27b‐3p
MAP3K2	MiR‐7‐2‐3p	(MSK1)	MiR‐23b‐3p
			MiR‐7‐2‐3p
MAP3K1	MiR‐7‐2‐3p		MiR‐205‐3p
			MiR‐25‐3p
MAP2K7	MiR‐27b‐3p		MiR‐106b‐3p

## 4 Discussion

**FIGURE 6:**
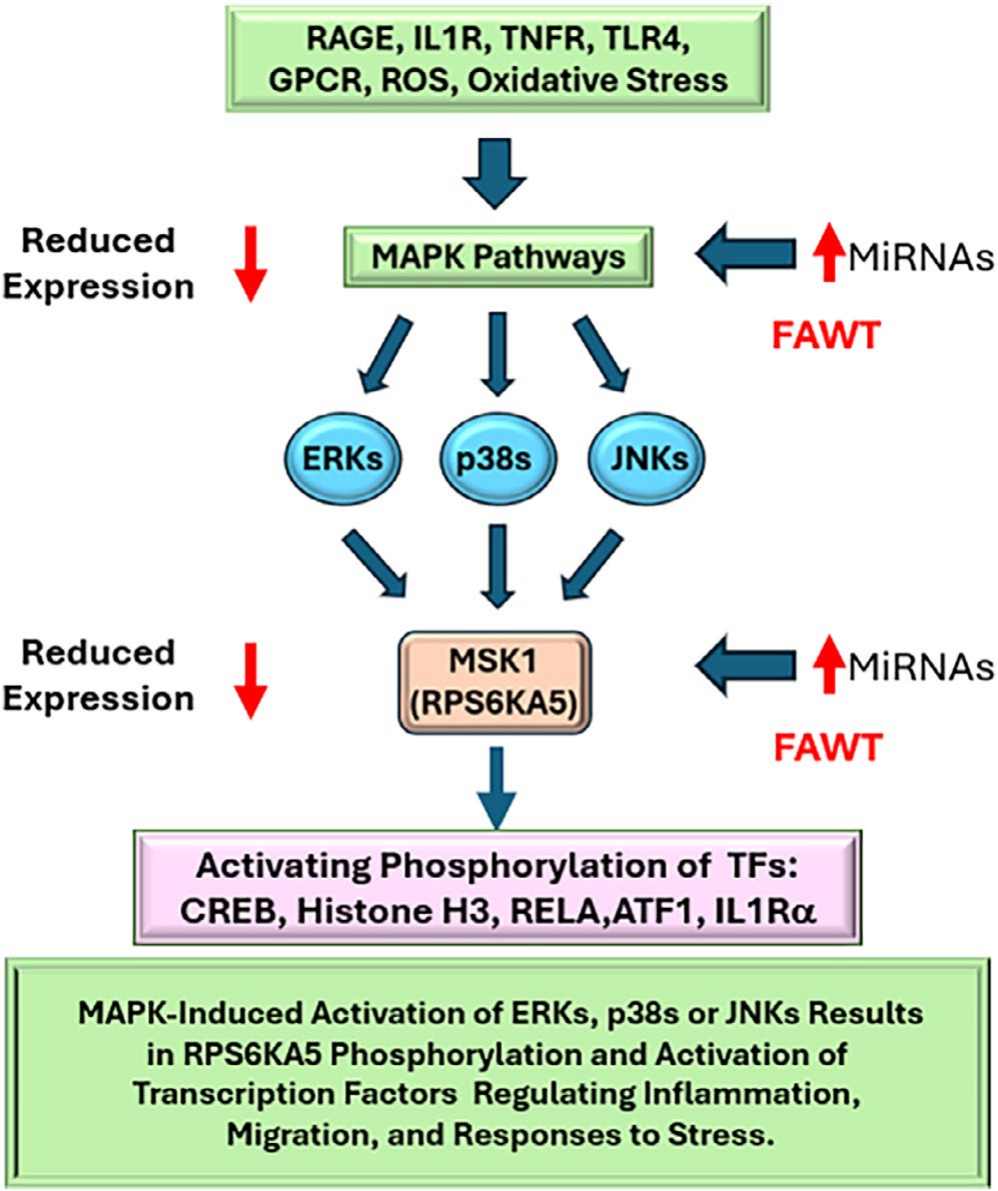
In the DFU, KCs sense an elevated inflammatory environment through cell surface receptors. Receptor (RAGE, IL1R, TNFR, TLR4, etc.) activation initiates signalling through MAPK pathways. These pathways culminate in the activation of ERKs, p38 MAPKs and/or SAPK/JNKs, which can activate RSK1 (RPS6KA1) and MSK1 (RPS6KA5) by phosphorylating multiple sites. MSK1 then phosphorylates specific sets of transcription factors (TFs) which regulate the expression of genes, whose products regulate inflammation, migration or responses to stress (ROS or genotoxicity). MSK1 also has several autophosphorylation sites that are regulated by cofactor associations and structural changes. In the RPPA data, we observed decreased phosphorylation of Ser 380 in MSK1, which is an indication of reduced ERK1/2 and p38a activity.

Multiple MiRNAs targeting RPS6KA5 (MSK1), MAP3K5 (ASK1), MAPK14 and MAP2K4 suggest decreased expression and lower protein levels. Proteomic analysis by RPPA identified significantly decreased levels of phosphorylated SAPK/JNK T183/Y185 and phosphorylated p38 MAPK T180/Y182 proteins. Taken together, these data suggest FAWT, by altering MiRNA expression, may have resulted in decreased p38 MAPKs and JNKs mediated activation of downstream kinases, RSK1 and MSK1, limiting these kinases' impact on transcription factor activation. RSK1 and MSK1 are both regulated through p38 MAPK signalling and decreased MSK1 would result in decreased phosphorylation of TFs that induced proinflammatory gene expression, lessening inflammation and promotion of proliferation or altering other cellular functions that could support improved healing. A simplified diagram of this impact of FAWT on MAPK‐targeting MiRNA expression signalling, the impact on MSK1 and its capacity to induce expression/activation of proinflammatory TFs is depicted in Figure 6. The impact of decreased upstream signalling and the MiRNAs targeting RPS6KA5 and RPS6KA1 suggests that FAWT alters the proinflammatory environment in DFU KC through increased expression of MiRNAs. Reduced MAPK activity leads to decreased expression of regulatory TFs.

KCs contribute to the proinflammatory wound environment influencing other immune cells through their release of chemokines and cytokines. In addition to the impact of FAWT on methylation in MiRNAs targeting MAPKs, FAWT decreased methylation of MiR‐4259 and MiR‐7‐2. These MiRNAs target and inhibit lactate dehydrogenase A (LDHA) protein expression (Supporting Information Table F). KCs are the major sources of lactate production in the epidermis. Lactate provides a critical gatekeeper function that may regulate the extracellular proinflammatory response of KCs, provided by activated neutrophils and macrophages [30].

KCs rely heavily on ATP to fuel proliferation of basal cells and to support migration of KCs to close the wound [31, 32]. In DFUs, chronic exposure to increasing levels of glucose and ROS production may result in increased mitochondrial dysfunction (decreasing inner mitochondrial membrane potential, DNA damage and altered mitochondrial functions) which lessens the production of ATP, critical for KC migration [33, 34]. Mitochondrial dysfunction contributes to diabetic complications and increased flux of pyruvate through LDHA results in the increased release of lactate into the wound bed [35]. With increased expression of MiRNAs inhibiting LDHA protein levels, the extracellular levels of lactate may decrease, lowering the potential for a proinflammatory response by neutrophils and macrophages.

FAWT treatment decreased methylation in regulatory sequences of MiR‐4259 and MiR‐7‐2, which may have increased their expression and depressed expression of LDHA. Thus, FAWT may lower the proinflammatory environment through a lessening of lactate synthesis and release into the wound bed. FAWT may decrease KC responses to the inflammatory environment by decreasing MAPK activation of proinflammatory TFs while also reducing the proinflammatory environment of the DFU.

The MAPKs, potentially targeted by FAWT‐induced MiRNAs, coalesce by lowering ERK and p38 MAPK activation of RSK1 and MSK1. This is reflected in the FAWT‐induced decreased activating phosphorylation of p38 MAPK in the RPPA analysis. There were multiple (7) MiRNAs targeting RPS6KA5, while only one, MiR‐93‐3p, might bind to RPS6KA1 and decrease RSK1 expression [36].

Combinatorial targeting, multiple MAPKs targeting one transcript, allows cells to fine tune responses [37]. Often, a predominant MiRNA significantly influences target mRNA translation, and this effect is potentiated by other targeting MiRNAs. This combinatorial effect allows for lower concentrations of each to exert a combined impact on target mRNA expression. While we only observed MiR‐93‐3p as possibly targeting RSP6KA1, there may have been some below our 90% cutoff that could impact expression. We did observe decreased methylation in MiRNAs targeting MAP2K4 and to a lesser extent MAPK14 (p38a) mRNA suggesting that FAWT resulted in a coordinated regulation and decreased downstream signalling from cell surface receptors to p38 MAPKs, and perhaps ERK1/2 and JNKs [38]. Decreased MSK1 phosphorylation resulting from impaired JNK, p38 MAPK and ERK1/2 activation could result in dysregulated phosphorylation of histone H3, CREB, NFKB and ATF1, which decreases proinflammatory gene expression. Activation of MSK‐1 initiates a cascade of signalling pathways involved in the early phases of the healing process and induces the production of IL‐10, which can repress proinflammatory signalling [39].

In review, there are multiple proteomic and genomic factors which have provided a unique perspective for opportunities to modulate a generally hostile extracellular DFU KC environment. Here, FAWT treatment may have promoted expression of MiRNAs which together resulted in significantly decreased expression of MAPKs, compromising the major signalling pathway from TLR4, RAGE, IL1R and so forth, that induces inflammation. FAWT decreased inflammation in DFU KC and promoted the recovery of critical DFU healing processes.

## 5 Summary

This preliminary study suggests that FAWT may prove to be an effective treatment for the management of chronic DFU by limiting impaired DFU healing and promoting wound re‐epithelialization. In the proinflammatory DFU, KC cell surface receptors (TNFRA, ILRs, TLRs, RAGE, etc.) are chronically activated under insult by multiple potent proinflammatory ligands. Study data provided evidence of FAWT upregulating the expression of MiRNAs that, in concert, may result in depression of proinflammatory KC signalling. These MiRNAs target MAPKs that direct signalling downstream to a group of three critical signalling nodes, ERKs, p38 MAPKs and JNKs, that form a critical MAPK nexus. Differential regulation of the upstream MAPK pathways may regulate the activity of RSK1 and MSK1. MSK1 is a ’Signal Processor’ that directs activation of TFs to regulate the expression of genes related to inflammation, stress responses, cell death/survival and chromatin remodelling. Limiting receptor signalling through MAPKs to decrease inflammasome formation reduced KC cellular expression of inflammatory mediators HMGB1 and Il‐1B, in DFU KCs. MiRNAs were also identified that target proteins (TRAF6, TAK1, IKKB) which are members of the TLR4 and RAGE signalling paths to MAPKs.

**FIGURE 7:**
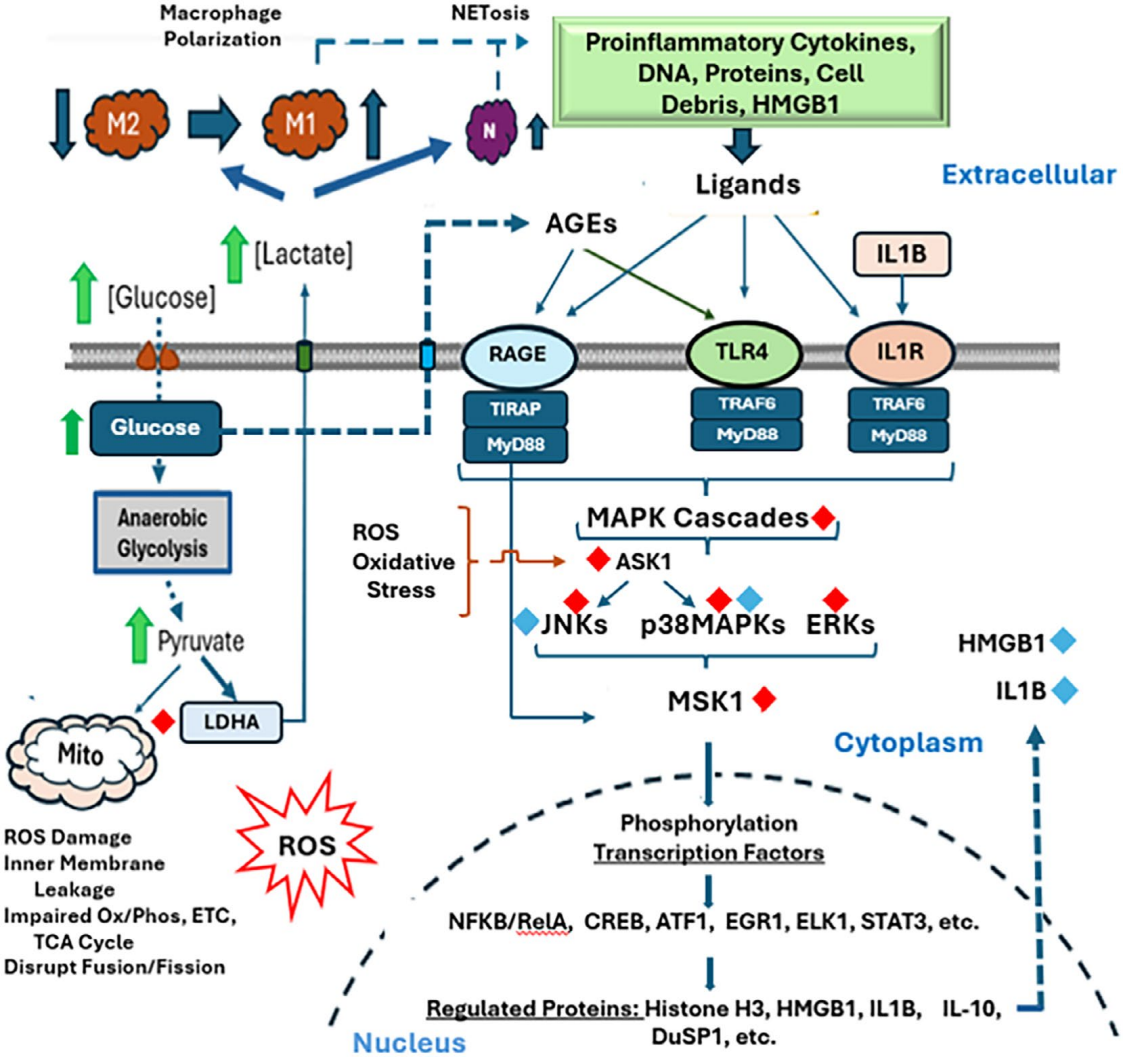
Diagram of signalling pathways activated in response to the proinflammatory ligands that induce receptor signalling through the MAPK Cascade in KC. Red diamonds (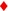) depict proteins that may be impacted through FAWT‐induced expression of targeting MiRNAs. Activated signalling proteins and end products of MSK1 activation IL1B and HMGB1, Blue (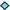), were significantly depressed following FAWT suggested up‐stream and down‐stream signalling through MSK1, a major regulatory hub controlling proinflammatory gene expression through transcription factor activation, may contribute to improved wound healing.

The observed impact of FAWT on DNA methylation provided additional pathways for the increased expression of several MiRNAs targeting multiple regulatory proteins (MAPKs, TRAF6, LDHA). While MAPK signalling through ERKs and p38 MAPKs activate MSK1, increased signalling through JNKs and p38 MAPKs also results in RSK1 activation [40]. These results suggest that FAWT lessened MAPK signalling downstream from, RAGE, TLR4 or IL1R, resulting in lowering ERK‐, JNK‐ and p38‐induced gene expression and the activation of proinflammatory TFs, perhaps by MSK1 or RSK1. Impaired signalling through these kinases alters gene expression, including HMGB1 and pro‐IL‐1B. FAWT may also be affecting immune cells, resulting in reduced levels of endogenous DAMPs (e.g., HMGB1) or lactate, decreasing the proinflammatory environment of the wound bed. These results lend support to the hypothesis that FAWT lessens the DFU KC proinflammatory response and its contribution to the proinflammatory environment. FAWT lessened TLR4 and RAGE inflammatory responses in DFU KC through regulation of MiRNAs which also facilitate KC proliferation and migration for wound closure. The apparent net result of FAWT is to lessen chronic DFU inflammation, allowing KCs, neutrophils and macrophages to stabilise and better support wound closure (Figure 7).

This exploratory RCT was designed to evaluate the merits of a folate‐supplemented chronic DFU treatment and identify potential mechanisms of action. However, there are several weaknesses of this clinical study. A larger, more powered study of this positive response of chronic DFUs to FAWT is required. Limitations inherent in this study include the small number of subjects in each group, which means any significant differences between Control and FAWT must be considered against the sample size. Statistical analysis of the RPPA data identified significantly (*p* < 0.05) depressed levels of HMGB1, IL‐1B, activated p38 MAPK and activated JNK3. Supporting methylation data showed that the mRNA for ASK1 (MAP3K5), RPS6KA1 and RPS6KA5 may be targeted by MiRNAs. While we have used stringent cut‐offs (greater than 90% probability) to identify potential MiRNA target mRNA interactions and employed multiple analytical algorithm programs, there is a need for clinical validation using the RPPA platform for protein quantitation and RT‐PCR or high‐throughput mRNA sequencing protocols to assess MiRNA‐mediated gene regulation. Additionally, transcription factor profiling is needed to assess the downstream impact of MSK‐1 on inflammation, migration, EMT‐MET and cell death pathways. Finally, an assessment of chromatin structure and histone modifications would be of interest to investigate how the methyl‐donating folinic acid resulted in demethylated regions following FAWT. Our study observations disclosed that molecular and cellular pathologies inherent with chronic DFUs may be surmounted through epigenomic regulation of MAPK signalling by FAWT. Additional proteogenomic investigations to validate these encouraging clinical findings are needed to provide additional foundation for our hypothesis.

Our documentation of significantly reduced levels of HMGB1 by FAWT in DFU KCs supports observations that multiple cell types involved in healing express receptors for HMGB1 [41]. HMGB1 is a predominant mediator of extracellular inflammation and promotes sustained inflammatory signalling in KCs and fibroblasts. High levels of HMGB1 inhibit collagen deposition and tensile strength in non‐healing wounds that may be reversed by decreasing HMGB1 levels [42]. In the non‐healing wound, increased proinflammatory activity of HMGB1 is mediated by its interactions with cytokines that set an exponentially increased inflammatory response that we see in DFUs [43]. Our observed decreased HMGB1 activity following FAWT suggests that, with lower HMGB1 levels, KCs in the DFU may not experience impaired proliferation and migration for wound closure that is exacerbated by cytokine induction. These observations are significant and provide a platform for continued research and development of FAWT. Supportive considerations from this study suggest that FAWT, if promoted, could have clinical value as an early interventional therapy for chronic DFUs that could provide benefit for the economic burden of DFU care. This is the first RCT to report on the use of a topical folic acid treatment for the impaired healing of DFUs. Our study results have enhanced our elucidation of proteogenomic signalling mechanisms that may support impaired healing of chronic DFUs and the corrective processes required to effectively promote DFU re‐epithelialization. Additionally, these early results also suggest that future research into folate metabolism may be identified as a therapeutic approach for other complications of type 2 diabetes or other proinflammatory diseases.

## Funding

This work was supported by Medline Industries.

## Disclosure

The content of this report is solely the responsibility of the authors and does not necessarily represent the official views of the US Department of Veterans Affairs or the United States Government.

## Conflicts of Interest

The authors declare no conflicts of interest.

## Supporting information


**Data S1:** wrr70141‐sup‐0001‐Supinfo.docx.

## Data Availability

The data that support the findings of this study are available on request from the corresponding author. The data are not publicly available due to privacy or ethical restrictions.

## References

[1] D. G. Armstrong , T. W. Tan , A. J. M. Boulton , and S. A. Bus , “Diabetic Foot Ulcers: A Review,” Journal of the American Medical Association 330, no. 1 (2023): 62–75, 10.1001/jama.2023.10578. 37395769 PMC10723802

[2] C. Lin , J. Liu , and H. Sun , “Risk Factors for Lower Extremity Amputation in Patients With Diabetic Foot Ulcers: A Meta‐Analysis,” PLoS One 15, no. 9 (2020): e0239236, 10.1371/journal.pone.0239236. 32936828 PMC7494323

[3] R. Hu , C. Q. Xia , E. Butfiloski , and M. Clare‐Salzler , “Effect of High Glucose on Cytokine Production by Human Peripheral Blood Immune Cells and Type I Interferon Signaling in Monocytes: Implications for the Role of Hyperglycemia in the Diabetes Inflammatory Process and Host Defense Against Infection,” Clinical Immunology 195 (2018): 139–148, 10.1016/j.clim.2018.06.003. 29894743 PMC6119493

[4] G. D. Hoke , C. Ramos , N. N. Hoke , M. C. Crossland , L. G. Shawler , and J. V. Boykin , “Atypical Diabetic Foot Ulcer Keratinocyte Protein Signaling Correlates With Impaired Wound Healing,” Journal Diabetes Research 2016 (2016): 1586927, 10.1155/2016/1586927. PMC5093264 27840833

[5] J. V. Boykin, Jr. , G. D. Hoke , C. R. Driscoll , and B. S. Dharmaraj , “High‐Dose Folic Acid and Its Effect on Early Stage Diabetic Foot Ulcer Wound Healing,” Wound Repair and Regeneration 28, no. 4 (2020): 517–525, 10.1111/wrr.12804. 32141182

[6] L. K. Park , A. G. Maione , A. Smith , et al., “Genome‐Wide DNA Methylation Analysis Identifies a Metabolic Memory Profile in Patient‐Derived Diabetic Foot Ulcer Fibroblasts,” Epigenetics 9, no. 10 (2014): 1339–1349. 25437049 10.4161/15592294.2014.967584 PMC4622843

[7] Z. Luka , S. Pakhomova , L. V. Loukachevitch , M. E. Newcomer , and C. Wagner , “Folate in Demethylation: The Crystal Structure of the Rat Dimethylglycine Dehydrogenase Complexed With Tetrahydrofolate,” Biochemical and Biophysical Research Communications 449, no. 4 (2014): 392–398, 10.1016/j.bbrc.2014.05.064. 24858690 PMC4113215

[8] A. Cianciulli , R. Salvatore , C. Porro , T. Trotta , and M. A. Panaro , “Folic Acid Is Able to Polarize the Inflammatory Response in LPS Activated Microglia by Regulating Multiple Signaling Pathways,” Mediators of Inflammation 2016 (2016): 5240127, 10.1155/2016/5240127. 27738387 PMC5055986

[9] L. Jian , Y. Cao , and Y. Zou , “Dermal‐Epidermal Separation by Heat,” Methods in Molecular Biology 2109 (2020): 23–25, 10.1007/7651_2019_270. 31792755

[10] A. Byron , S. Bernhardt , B. Ouine , et al., “Integrative Analysis of Multi‐Platform Reverse‐Phase Protein Array Data for the Pharmacodynamic Assessment of Response to Targeted Therapies,” Scientific Reports 10, no. 1 (2020): 21985. 33319783 10.1038/s41598-020-77335-0 PMC7738515

[11] R. T. Barfield , V. Kilaru , A. K. Smith , and K. N. Conneely , “CpGassoc: An R Function for Analysis of DNA Methylation Microarray Data,” Bioinformatics 28, no. 9 (2012): 1280–1281, 10.1093/bioinformatics/bts124. 22451269 PMC3577110

[12] W. Zhou , T. J. Triche , P. W. Laird , and H. Shen , “SeSAMe: Reducing Artifactual Detection of DNA Methylation by Infinium BeadChips in Genomic Deletions,” Nucleic Acids Research 46, no. 20 (2018): e123, 10.1093/nar/gky691. 30085201 PMC6237738

[13] G. Zhou , O. Soufan , J. Ewald , R. E. W. Hancock , N. Basu , and J. Xia , “NetworkAnalyst 3.0: A Visual Analytics Platform for Comprehensive Gene Expression Profiling and Meta‐Analysis,” Nucleic Acids Research 47, no. W1 (2019): W234–W241. 30931480 10.1093/nar/gkz240 PMC6602507

[14] W. Liu and X. Wang , “Prediction of Functional microRNA Targets by Integrative Modeling of microRNA Binding and Target Expression Data,” Genome Biology 20 (2019): 18. 30670076 10.1186/s13059-019-1629-z PMC6341724

[15] Y. Chen and X. Wang , “miRDB: An Online Database for Prediction of Functional microRNA Targets,” Nucleic Acids Research 48, no. D1 (2020): D127–D131. 31504780 10.1093/nar/gkz757 PMC6943051

[16] V. Agarwal , G. W. Bell , J. W. Nam , and D. P. Bartel , “Predicting Effective microRNA Target Sites in Mammalian mRNAs,” eLife 4 (2015): e05005, 10.7554/eLife.05005. 26267216 PMC4532895

[17] S. Tastsoglou , G. Skoufos , M. Miliotis , et al., “DIANA‐miRPath v4.0: Expanding Target‐Based miRNA Functional Analysis in Cell‐Type and Tissue Contexts,” Nucleic Acids Research 51 (2023): W154–W159, 10.1093/nar/gkad431. 37260078 PMC10320185

[18] F. Kern , E. Aparicio‐Puerta , Y. Li , et al., “miRTargetLink 2.0—Interactive miRNA Target Gene and Target Pathway Networks,” Nucleic Acids Research 49 (2021): W409–W416, 10.1093/nar/gkab297. 34009375 PMC8262750

[19] S. H. Kim , J. Lee , J. Jung , et al., “Interruption of p38^MAPK^‐MSK1‐CREB‐MITF‐M Pathway to Prevent Hyperpigmentation in the Skin,” International Journal of Biological Sciences 20, no. 5 (2024): 1688–1704, 10.7150/ijbs.93120. 38481807 PMC10929196

[20] A. M. Owen , L. Luan , K. R. Burelbach , et al., “MyD88‐Dependent Signaling Drives Toll‐Like Receptor‐Induced Trained Immunity in Macrophages,” Frontiers in Immunology 13 (2022): 1044662, 10.3389/fimmu.2022.1044662. 36439136 PMC9692127

[21] M. R. Dasu and I. Jialal , “Amelioration in Wound Healing in Diabetic Toll‐Like Receptor‐4 Knockout Mice,” Journal of Diabetes and Its Complications 27, no. 5 (2013): 417–421, 10.1016/j.jdiacomp.2013.05.002. 23773694 PMC3770740

[22] G. Stelzer , R. Rosen , I. Plaschkes , et al., “The GeneCards Suite: From Gene Data Mining to Disease Genome Sequence Analyses,” in Current Protocols in Bioinformatics, ed. M. Safran, N. Rosen, M. Twik, et al., vol. 54 ( John Wiley & Sons, Inc., 2016), 1.30.1–1.30.33, 10.1002/cpbi.5. 27322403

[23] M. Safran , N. Rosen , M. Twik , et al., “The GeneCards Suite Chapter,” in Chapter Practical Guide to Life Science Databases (John Wiley & Sons, Inc., 2022), 27–56.

[24] M. Cargnello and P. P. Roux , “Activation and Function of the MAPKs and Their Substrates, the MAPK‐Activated Protein Kinases,” Microbiology and Molecular Biology Reviews 75, no. 1 (2011): 50–83, 10.1128/MMBR.00031-10 Erratum in: Microbiology and Molecular Biology Reviews 2012 Jun;76(2):496. 21372320 PMC3063353

[25] M. Katz , I. Amit , and Y. Yarden , “Regulation of MAPKs by Growth Factors and Receptor Tyrosine Kinases,” Biochimica et Biophysica Acta 1773, no. 8 (2007): 1161–1176, 10.1016/j.bbamcr.2007.01.002. 17306385 PMC2758354

[26] J. Deguine and G. M. Barton , “MyD88: A Central Player in Innate Immune Signaling,” F1000Prime Reports 6 (2014): 97, 10.12703/P6-97. 25580251 PMC4229726

[27] A. Gugliandolo , S. Silvestro , C. Sindona , P. Bramanti , and E. Mazzon , “MiRNA: Involvement of the MAPK Pathway in Ischemic Stroke. A Promising Therapeutic Target,” Medicina 57, no. 10 (2021): 1053, 10.3390/medicina57101053. 34684090 PMC8539390

[28] Y. Li , X. Chen , and C. Lu , “The Interplay Between DNA and Histone Methylation: Molecular Mechanisms and Disease Implications,” EMBO Reports 22, no. 5 (2021): e51803, 10.15252/embr.202051803. 33844406 PMC8097341

[29] M. Milacic , D. Beavers , P. Conley , et al., “The Reactome Pathway Knowledgebase 2024,” Nucleic Acids Research 52 (2024): D672–D678, 10.1093/nar/gkad1025. PMC10767911 37941124

[30] D. Ruan , T. Hu , X. Yang , X. Mo , and Q. Ju , “Lactate in Skin Homeostasis: Metabolism, Skin Barrier, and Immunomodulation,” Frontiers in Immunology 16 (2025): 1510559, 10.3389/fimmu.2025.1510559. 40046050 PMC11879785

[31] S. Prahl , T. Kueper , T. Biernoth , et al., “Aging Skin Is Functionally Anaerobic: Importance of Coenzyme Q10 for Anti Aging Skin Care,” BioFactors 32, no. 1–4 (2008): 245–255, 10.1002/biof.5520320129. 19096122

[32] W. Zwerschke , S. Mazurek , P. Stöckl , E. Hütter , E. Eigenbrodt , and P. Jansen‐Dürr , “Metabolic Analysis of Senescent Human Fibroblasts Reveals a Role for AMP in Cellular Senescence,” Biochemical Journal 376 (2003): 403–411, 10.1042/bj20030816. 12943534 PMC1223775

[33] P. González , P. Lozano , G. Ros , and F. Solano , “Hyperglycemia and Oxidative Stress: An Integral, Updated and Critical Overview of Their Metabolic Interconnections,” International Journal of Molecular Sciences 24, no. 11 (2023): 9352, 10.3390/ijms24119352. 37298303 PMC10253853

[34] F. N. Iheagwam , A. J. Joseph , E. D. Adedoyin , O. T. Iheagwam , and S. A. Ejoh , “Mitochondrial Dysfunction in Diabetes: Shedding Light on a Widespread Oversight,” Pathophysiology 32, no. 1 (2025): 9, 10.3390/pathophysiology32010009. 39982365 PMC12077258

[35] A. D. Wise , E. G. TenBarge , J. D. C. Mendonça , et al., “Mitochondria Sense Bacterial Lactate and Drive Release of Neutrophil Extracellular Traps,” Cell Host & Microbe 33, no. 3 (2025): 341–357.e9, 10.1016/j.chom.2025.02.003. 40020664 PMC11955204

[36] A. M. Gurtan and P. A. Sharp , “The Role of miRNAs in Regulating Gene Expression Networks,” Journal of Molecular Biology 425, no. 19 (2013): 3582–3600, 10.1016/j.jmb.2013.03.007. 23500488 PMC3757117

[37] M. Peter , “Targeting of mRNAs by Multiple miRNAs: The Next Step,” Oncogene 29 (2010): 2161–2164, 10.1038/onc.2010.59). 20190803

[38] H. Sattarifard , A. Safaei , E. Khazeeva , M. Rastegar , and J. R. Davie , “Mitogen‐ and Stress‐Activated Protein Kinase (MSK1/2) Regulated Gene Expression in Normal and Disease States,” Biochemistry and Cell Biology 101, no. 3 (2023): 204–219, 10.1139/bcb-2022-0371. 36812480

[39] K. M. Reyskens and J. S. Arthur , “Emerging Roles of the Mitogen and Stress Activated Kinases MSK1 and MSK2,” Frontiers in Cell and Development Biology 10, no. 4 (2016): 56, 10.3389/fcell.2016.00056. PMC4901046 27376065

[40] C. Hauge and M. Frödin , “RSK and MSK in MAP Kinase Signaling,” Journal of Cell Science 119, no. Pt 15 (2006): 3021–3023, 10.1242/jcs.02950. 16868029

[41] E. Ranzato , S. Martinotti , M. Pedrazzi , and M. Patrone , “High Mobility Group Box Protein‐1 in Wound Repair,” Cells 1, no. 4 (2012): 699–710, 10.3390/cells1040699. 24710526 PMC3901153

[42] Q. Zhang , S. O'Hearn , S. L. Kavalukas , and A. Barbul , “Role of High Mobility Group Box 1 (HMGB1) in Wound Healing,” Journal of Surgical Research 176 (2012): 343–347, 10.1016/j.jss.2011.06.069. 21872885

[43] Y. Sha , J. Zmijewski , X. Zhiwei , and E. Abraham , “2008 HMGB1 Develops Enhanced Proinflammatory Activity by Binding to Cytokines,” Journal of Immunology 180 (2008): 2531–2537. 10.4049/jimmunol.180.4.2531 18250463

