# Supplementary material for: Folinic Acid Improves Healing of Diabetic Foot Ulcers
Source: Wound Repair Regen. 2026 Mar 7;34(2):e70141. doi: 10.1111/wrr.70141 (PMC12967698; doi:10.1111/wrr.70141)

**Supplementary Tables: A-G**

For the FAWT Post Diff. Scores, positive numbers represent increased fold changes in the level of miRNA methylation, suggesting decreased MiRNA expression, while FAWT Post Diff Scores, in parentheses, are for decreased methylation fold changes, suggesting increased expression of the MiRNA.

**Levels of validation include:**

**Strong Validation:** Signifies more than one analytical program identified an MiRNA as a target and that had indications of supporting functional analysis with the targeted mRNA. Functional analysis is mainly based up literature and the TarBase analytical platform ( [http://www.microrna.gr/tarbase](http://diana.imis.athena-innovation.gr/DianaTools/index.php?r=tarbase/index)), which is the largest available manually curated target database, indexing more than 65,000 miRNA-gene interactions. The database includes targets derived from specific, as well as high throughput experiments, such as microarrays and proteomics.

**Validated Targets:** Signifies those MiRNAs that were identified in more than one MiRNA analytical program based upon MiRNA Seed Sequence match with the targeted mRNA.

**Targets:** Signifies those MiRNAs that were identified in one analytical program based upon MiRNA Seed Sequence match with the targeted mRNA.

**Table A: MiR-27b-3p, MiR-8085-3p, MiR-23b-3p and MiR-7-2-3p**


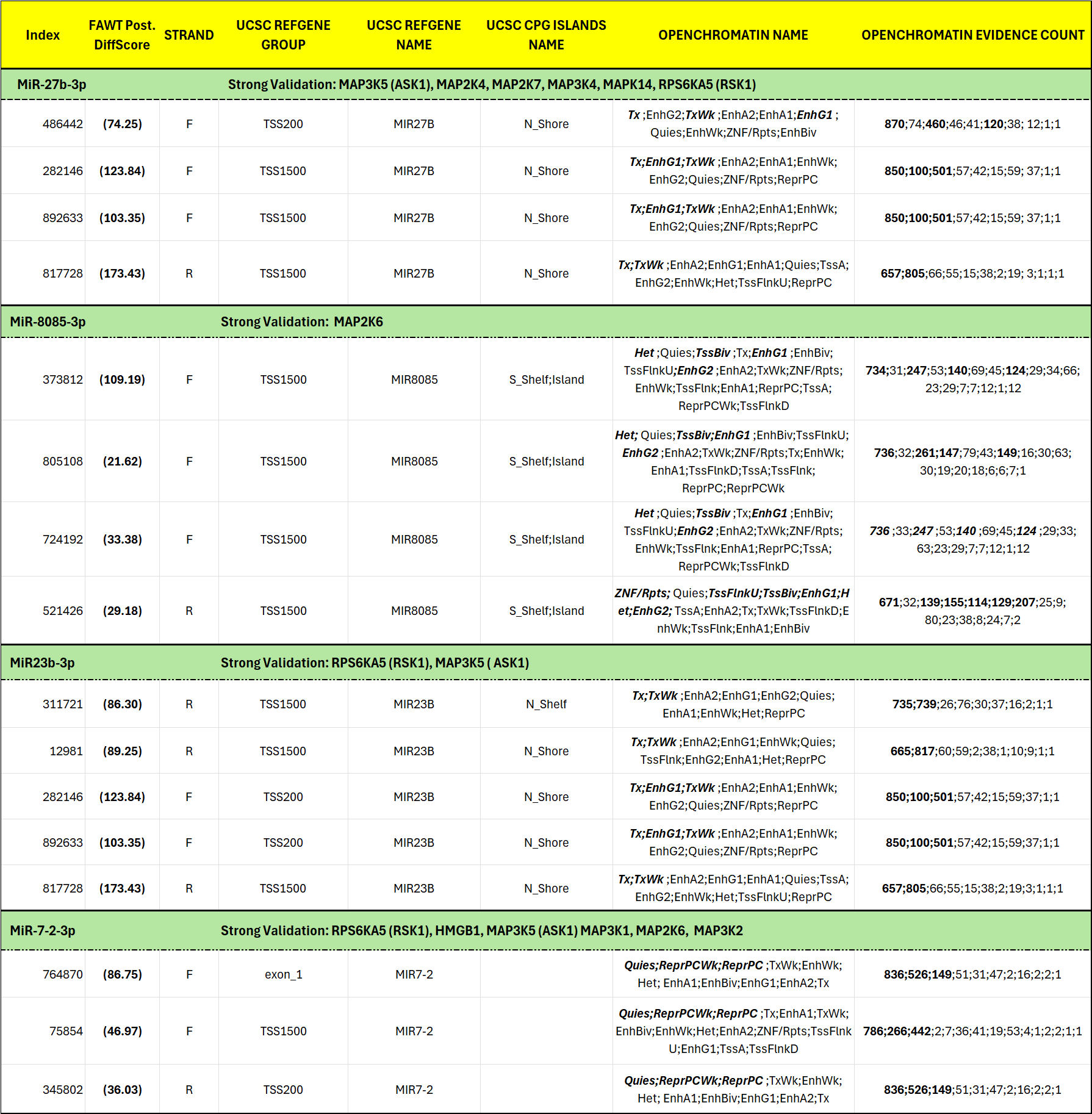


**Table B: MiR-9-3-3p, MiR2682-3p and MiR-205-3p**

**Table C: MiR-25-3p, MiR106b-3p, MiR-1298-3p, MiR-186-3p,**

**and MiR-4763-3p**


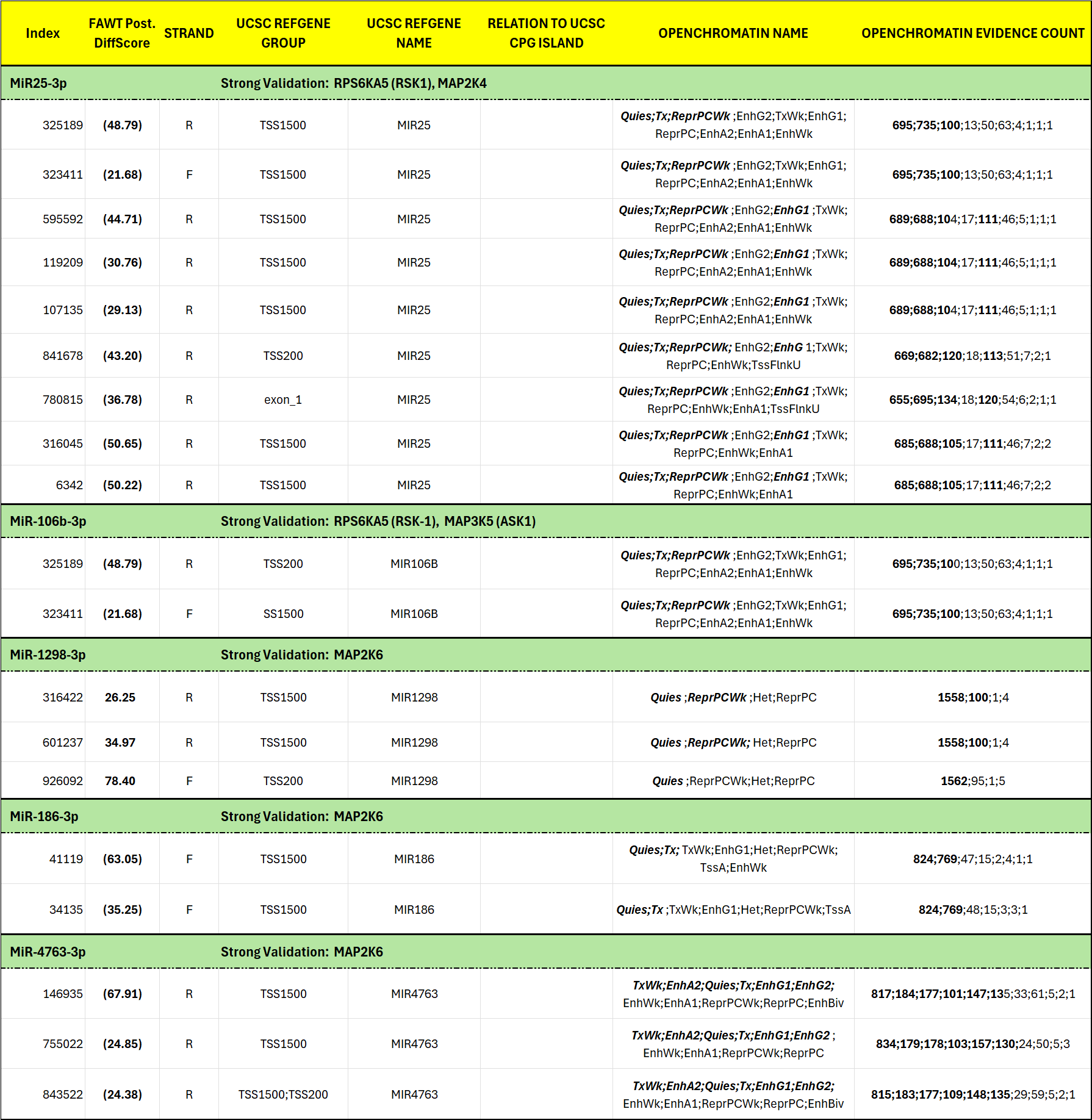


**Table D: MiR-Let-7B-5p and MiR-196A2**


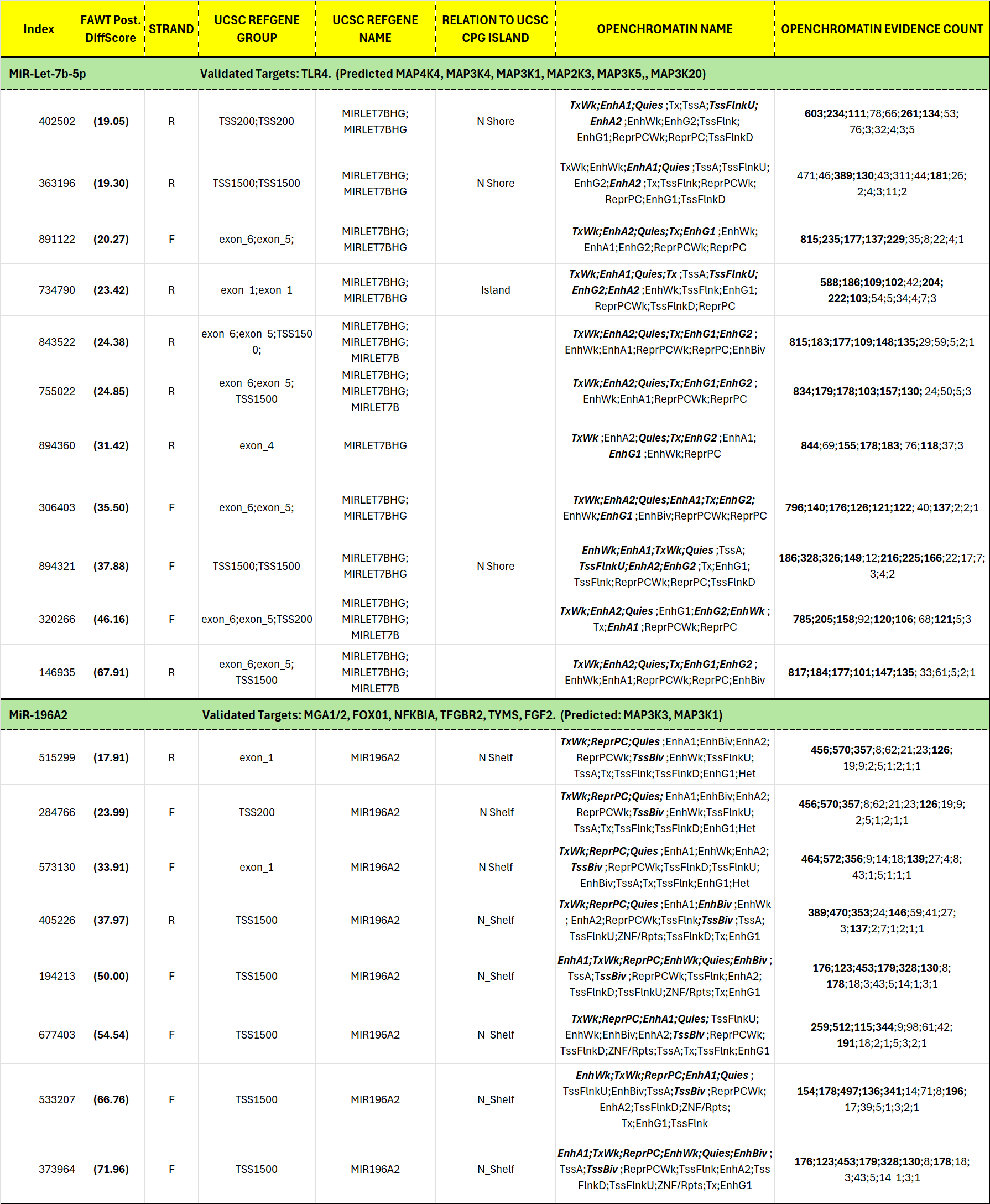


**Table E: MiR-141-3p and MiR-200c**


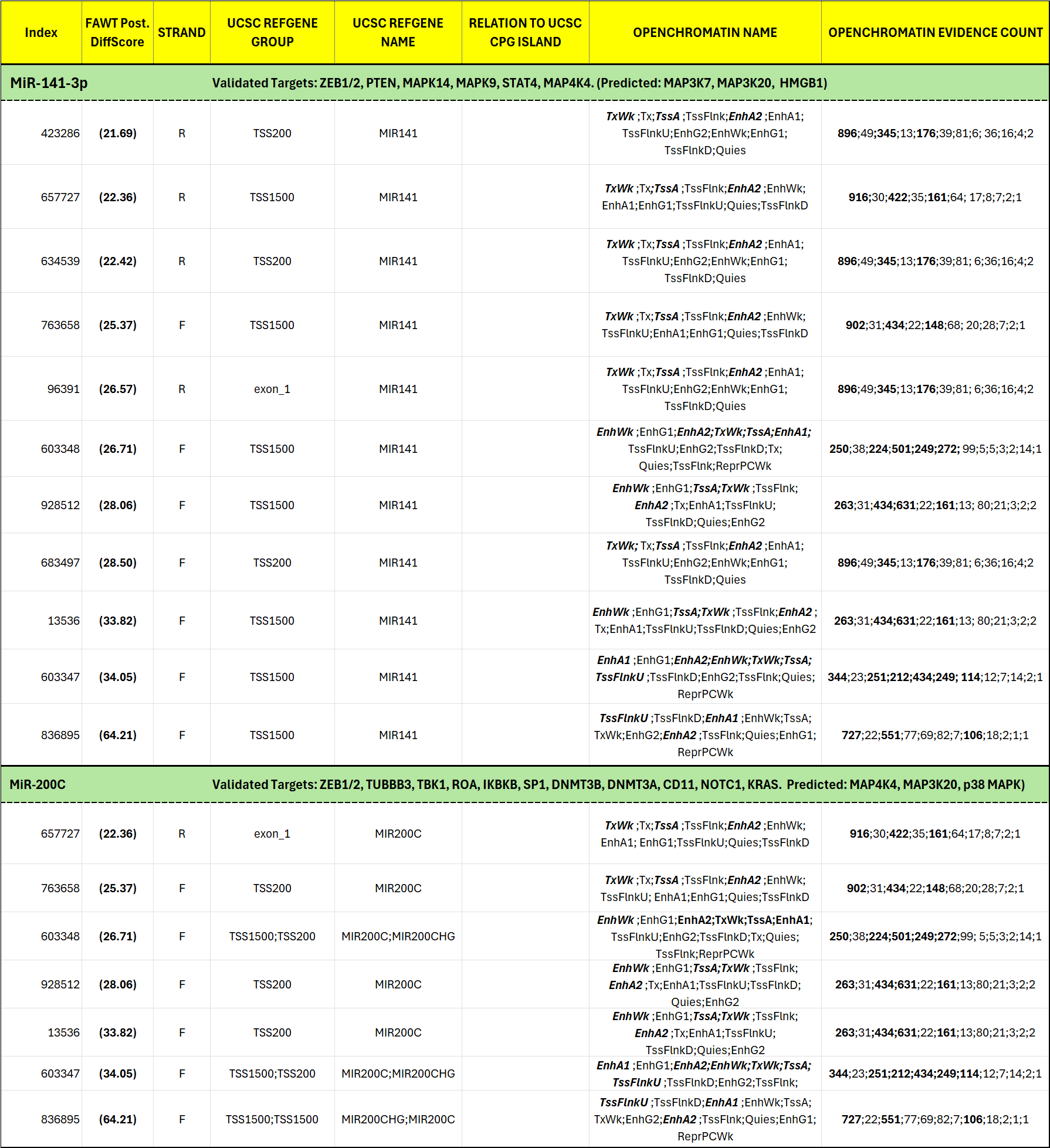


**Table F: MiR-24, MiR-4259-3p and MiR-33b-5p**


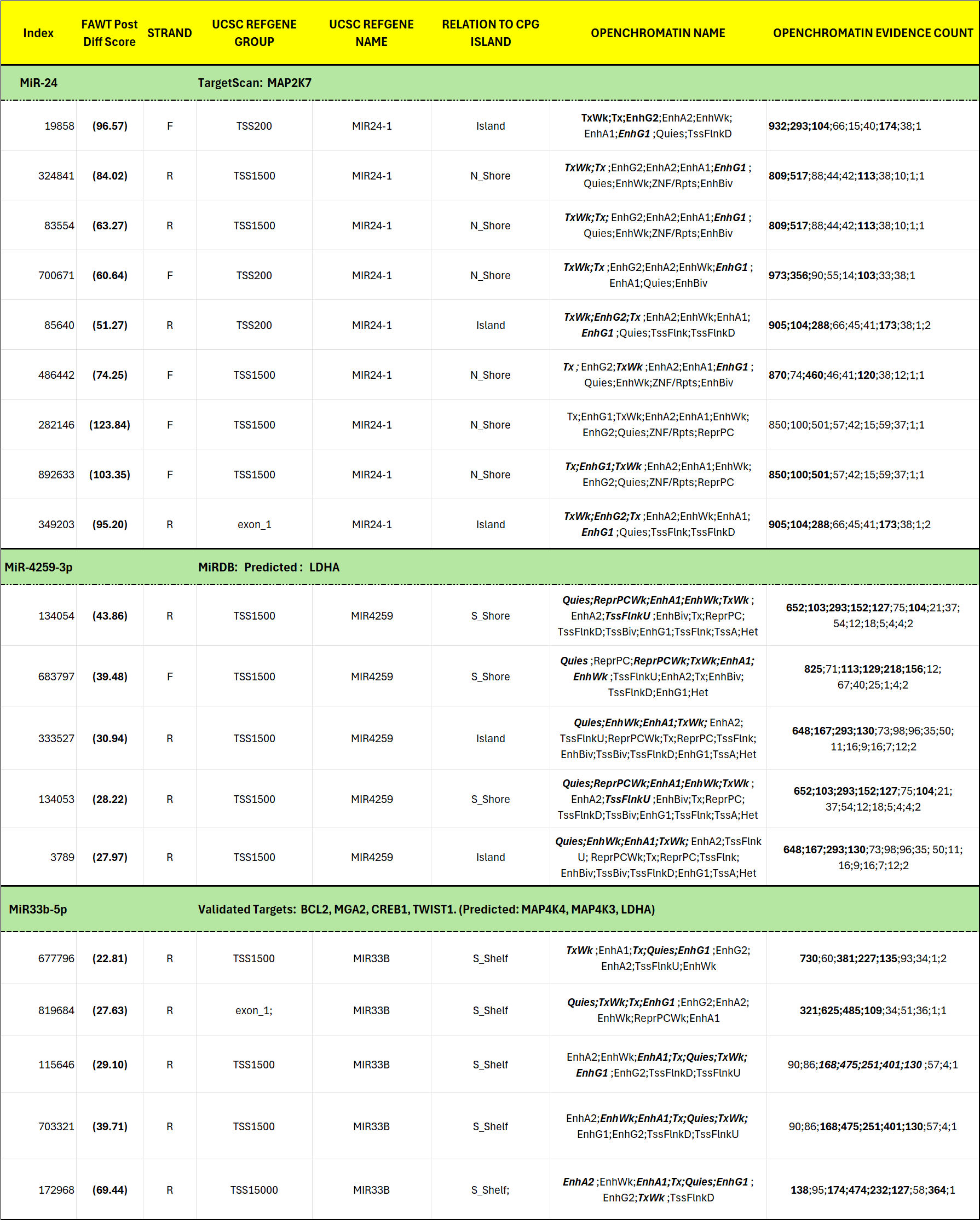


**Table G: MiR-93-3p**


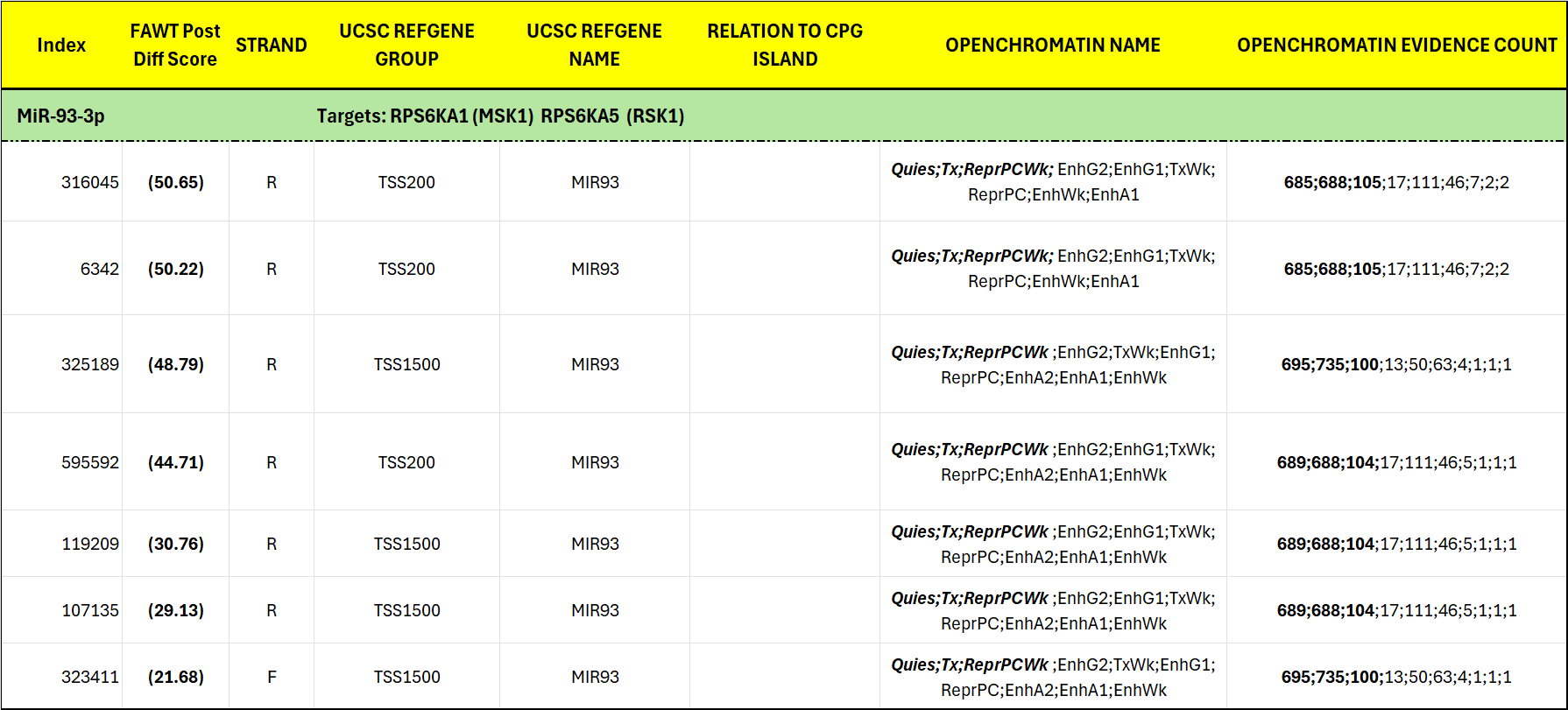

Supplement: Supplementary file 1 — Data S1: wrr70141‐sup‐0001‐Supinfo.docx. [file WRR-34-0-s001.docx]
